# On the Thermal Models for Resistive Random Access Memory Circuit Simulation

**DOI:** 10.3390/nano11051261

**Published:** 2021-05-11

**Authors:** Juan B. Roldán, Gerardo González-Cordero, Rodrigo Picos, Enrique Miranda, Félix Palumbo, Francisco Jiménez-Molinos, Enrique Moreno, David Maldonado, Santiago B. Baldomá, Mohamad Moner Al Chawa, Carol de Benito, Stavros G. Stavrinides, Jordi Suñé, Leon O. Chua

**Affiliations:** 1 Departamento de Electrónica y Tecnología de Computadores, Facultad de Ciencias, Universidad de Granada, Avd. Fuentenueva s/n, 18071 Granada, Spain; g2cordr@gmail.com (G.G.-C.); jmolinos@ugr.es (F.J.-M.); dmaldonado@ugr.es (D.M.); 2 Industrial Engineering and Construction Department, University of Balearic Islands, 07122 Palma, Spain; rodrigo.picos@uib.es (R.P.); carol.debenito@uib.es (C.d.B.); 3 Department Enginyeria Electrònica, Universitat Autònoma de Barcelona, Edifici Q., 08193 Bellaterra, Spain; enrique.miranda@uab.cat (E.M.); jordi.sune@uab.cat (J.S.); 4 Consejo Nacional de Investigaciones Científicas y Técnicas (CONICET), Godoy Cruz 2290, Buenos Aires C1425FQB, Argentina; felix.palumbo@conicet.gov.ar; 5 UJM-St-Etienne, CNRS, Laboratoire Hubert Curien UMR 5516, Institute of Optics Graduate School, University Lyon, F-42023 St-Etienne, France; enrique.manuel.moreno.perez@univ-st-etienne.fr; 6 Unidad de Investigación y Desarrollo de las Ingenierías (UIDI), Facultad Regional Buenos Aires, Universidad Tecnológica Nacional, Medrano 951, Buenos Aires C1179AAQ, Argentina; sanboyeras@gmail.com; 7 Institute of Circuits and Systems, Technische Universität Dresden, 01062 Dresden, Germany; mohamad_moner.al_chawa@tu-dresden.de; 8 School of Science and Technology, Thermi University Campus, International Hellenic University, 57001 Thessaloniki, Greece; s.stavrinides@ihu.edu.gr; 9 Electrical Engineering and Computer Science Department, University of California, Berkeley, CA 94720-1770, USA; chua@berkeley.edu

**Keywords:** resistive memories, thermal model, heat equation, thermal conductivity, circuit simulation, compact modeling, resistive switching, nanodevices

## Abstract

Resistive Random Access Memories (RRAMs) are based on resistive switching (RS) operation and exhibit a set of technological features that make them ideal candidates for applications related to non-volatile memories, neuromorphic computing and hardware cryptography. For the full industrial development of these devices different simulation tools and compact models are needed in order to allow computer-aided design, both at the device and circuit levels. Most of the different RRAM models presented so far in the literature deal with temperature effects since the physical mechanisms behind RS are thermally activated; therefore, an exhaustive description of these effects is essential. As far as we know, no revision papers on thermal models have been published yet; and that is why we deal with this issue here. Using the heat equation as the starting point, we describe the details of its numerical solution for a conventional RRAM structure and, later on, present models of different complexity to integrate thermal effects in complete compact models that account for the kinetics of the chemical reactions behind resistive switching and the current calculation. In particular, we have accounted for different conductive filament geometries, operation regimes, filament lateral heat losses, the use of several temperatures to characterize each conductive filament, among other issues. A 3D numerical solution of the heat equation within a complete RRAM simulator was also taken into account. A general memristor model is also formulated accounting for temperature as one of the state variables to describe electron device operation. In addition, to widen the view from different perspectives, we deal with a thermal model contextualized within the quantum point contact formalism. In this manner, the temperature can be accounted for the description of quantum effects in the RRAM charge transport mechanisms. Finally, the thermometry of conducting filaments and the corresponding models considering different dielectric materials are tackled in depth.

## 1. Introduction

Resistive memories (also known as resistive random access memories or RRAMs) base their operation on resistive switching mechanisms to modulate their conductance in a non-volatile manner [1,2,3]. Their promising potential is subject to scrutiny both in academia and in industry. These devices could be alternatives to flash devices in applications related to their non-volatility, such as storage class memories [4]. RRAMs show short read/write times, good enough endurance and retention behavior, low power operation, CMOS technology compatibility and the possibility to be built on 3D stacks [1,2]. Apart from non-volatile memory circuits, where certain applications are currently on the market, RRAMs show great potential for cryptographic circuits due to their inherent stochasticity, which can be employed for the design of random number generators and physical unclonable functions [5,6,7,8,9]. Nevertheless, currently, the hottest application for these devices is linked to neuromorphic circuits [10,11,12,13,14,15]. RRAMs can mimic biological synapses within a fully compatible CMOS technology context to facilitate the fabrication of hardware neural networks. This approach allows the use of a lower number of electronic components (by means of RS device crossbars) and low power consumption [1,10,11,14,16] than the purely CMOS alternative to neuromorphic circuits firstly introduced by Mead in the late eighties [17]. The number of papers on this subject including resistive switching devices is growing by leaps and bounds. Nevertheless, different issues should be improved for these devices in order to overcome the difficulties connected to massive industrial use; among them, the following should be counted: great temperature sensitivity, manufacturing processes, variability and the lack of well-established electronic design automation (EDA) tools, including in the latter issue strategies for parameter extraction. In relation to these essential aspects, we will deal here with the device thermal description and its modeling from a circuit simulation perspective.

The development of RRAM simulation and modeling infrastructure is key to step forward into industrial mature applications. In this respect, in the memory realm, it is important to highlight that DRAM and NAND Flash technologies will continue to hold their ground and even advance despite the slowing of Moore’s Law in the short-medium term. Although a great number of papers have been published so far on modeling and simulation issues [18,19,20,21,22,23,24,25,26,27,28,29,30,31,32,33,34,35,36,37,38,39,40,41], there are many open questions that need to be addressed to improve RRAM position in the EDA context. One of the pressing modeling questions is connected with the device inherent stochasticity that produces cycle-to-cycle variability [19,42,43,44,45,46,47,48,49,50,51]. This type of variability has to be managed to achieve RRAM technological maturity; however, even if a variability reduction is achieved, taking into consideration its nature in the modeling is a must [52]. Another modeling battlefield lies in what is linked to thermal effects. It is known that most RS mechanisms are thermally activated [19,23,32,53,54,55,56,57]. The temperature evolution produced by Joule heating in device-relevant regions, where charge conduction is concentrated, determines the operation in many different types of RRAMs [18,19,38,53,58,59,60]. Consequently, an accurate thermal description is essential to implement both good RRAM physical simulators and compact models. It is also important to highlight that in cross-bar architectures, an optimum topology for hardware neural network implementation among other applications, the thermal evolution of one device might be influenced by neighbor cells. This effect, known as thermal crosstalk [60,61,62,63], has to be considered at the integration stage of chips based on RS devices.

Modeling of RRAM, as well as its physical simulation in general, shows notorious differences with respect to other electron devices. In this respect, in devices such as MOSFETs [64,65] (even multigate FETs [66]) or diodes [67,68], once a reasonable grid is established, the main differential equations are discretized and, in general, but for very scarce cases, convergence is searched for to obtain a solution. This is the main scheme to follow in drift-diffusion, hydrodynamic and Monte Carlo simulation approaches [69]. By contrast, in RRAMs the modeling paradigm is different due to the particularities of their operation. For the initial forming process (a pristine dielectric is assumed) and further set processes within RS cycles (in the common filamentary conduction regime, the case we will deal with henceforth), a conductive filament (CF) through the dielectric is formed which shorts the device electrodes and greatly reduce the device resistance [2,70]. Non-linear physical mechanisms (mostly thermally activated, following an Arrhenius’ equation relationship) come into play in a positive feedback loop that leads certain magnitudes, such as the temperature, to shoot up. This process has an obvious reflection at the experimental level, the current has to be limited to avoid the device hard breakdown and its consequent destruction. All this leads to the caveat that we have to deal with numerical divergence from the simulation viewpoint. The current is limited in the context of forming or set processes to avoid the device rupture; in this manner, an uncontrolled temperature rise and the corresponding CF quick growth is avoided because after a hard breakdown process RS operation does not hold. At the simulation level, a current limit is also needed. The reset process is also linked to RRAM simulation divergence. As the reset goes on, the CF shrinks and the same current is strangled in a CF narrow section. Hence, Joule heating produces a temperature increase at this CF narrowing. The thermally activated mechanisms in the CF narrowing surroundings rise exponentially and the CF rupture takes place. If the rupture is thermally controlled, as in many unipolar devices, it is based upon a thermal run-away process that leads to the CF destruction. In summary, for a correct RRAM simulation description (both at the device and circuit levels, in the latter case it would be a compact modeling approach), we have to face numerical divergence in addition to nonlinearity in some of the physical magnitudes at hand in one way or other. This means, in general, as the reader can imagine, a numerical nightmare. For instance, when you set up a limit for the compliance current, there might be an important difference between the iteration prior to achieving the current limit and the following step (once the compliance current has been exceeded) in terms of the CF size and temperature in the hottest spot. If you miss to stop the iterations just when the compliance current is reached, and for some reason you iterate once more within the positive feedback loop the device is going through, you can end up wondering what is the dielectric melting temperature.

There are different RRAM models in the literature, and most of them consider thermal effects in one way or another. Some assume a simplified version of the steady-state heat equation (HE); others account for a non-steady-state model where the heat capacitance is included. In general, the main differences are found in the boundary conditions formulation to describe the device physics in the context of the HE or an equivalent energy balance equation. In any case, all these models can be formulated using the standard description proposed by [71], where they fit naturally. By using this formulation, it is clear that all these devices can be considered as extended memristors, this being the most general class of memristor devices according to usual classification [72]. Notice that, from this point of view, RRAM devices can be used inside the memristor framework as circuital elements for purposes further than memory applications [73,74,75].

Different flavors of these thermal models have been reported; however, as far as we know, they have not been brought together under one roof yet as has been done, for example, with electrical models [41]. We do so here. In Section 2, we comment on the HE in the RRAM operation context, in particular, we explain 3D physical simulation and compact modeling focusing on thermal effects. Section 3 is devoted to the RRAM thermal description through a general memristor model; the RRAM quantum point contact modeling including thermal effects is unfolded in Section 4, and finally, the thermometry of conducting filaments and corresponding modeling is developed in Section 5.

## 2. Mathematical Description of RRAM Thermal Effects

### 2.1. Heat Equation

We start by taking the 3D heat equation in the device into consideration, Equation (1):

(1) $$\nabla \cdot \left(k_{th}\left(r\right)\nabla T\left(r,t\right)\right)+{\dot{e}}_{generated}\left(r\right)=\rho \left(r\right)c\left(r\right)\frac{\partial T\left(r,t\right)}{\partial t}$$

where *k_th_*—stands for the thermal conductivity (W/(m K)). This parameter depends on the temperature and geometry (assuming different material layers, which is the case for the usual RRAM architecture), *c*—stands for the specific heat or specific heat capacity (J/(kg K)). It is assumed that we are considering the specific heat capacity at constant pressure, which is why it is also denoted as c_p_, ρ—stands for the material density (kg/m^3^) and ${\dot{e}}_{generated}$—stands for the power density generated (rate of heat generation by means of Joule heating, per unit volume inside the system we are considering). It can be calculated as σ(***r***)E^2^(***r***), where σ(***r***) is the local electrical conductivity and E(***r***) is the local electric field [18,38]; i.e., the product of the field and the current density.

The RRAM thermal description requires the solution of this equation in the whole device active region. Nevertheless, in the compact modeling approach (CMA) some simplifying assumptions are made. Among others, we consider this equation in the region close to the conductive filament, where charge conduction takes place after a successful set or forming process (when the CF is created the device is said to be in the low resistance state, LRS, while if the CF is ruptured, after a successful reset process, the device resistance is much higher, the device enters in the high resistance state, HRS). If all the different device material layers are included (dielectric, possibly a multilayer stack, electrodes, etc.), the thermal conductivity, the density and specific heat of the different materials have to be considered [29,76,77].

A step forward consists in using the 1D HE version (a simplifying assumption that works well in many cases). If the x coordinate is assumed to be parallel to the CF longitudinal axis, from one of the dielectric-electrode interfaces to the other, and the CF is considered to be narrow enough to consider the same temperature in the transverse sections perpendicular to the x-axis; then, the HE could be written as follows:

(2) $$\frac{\partial }{\partial x}\left(k_{th}\left(x\right)\frac{\partial T\left(x,t\right)}{\partial x}\right)+{\dot{e}}_{generated}\left(x\right)=\rho \left(x\right)c\left(x\right)\frac{\partial T\left(x,t\right)}{\partial t}$$

This equation is most of the times solved within the device conductive filament, whose geometry is assumed to be a cylinder or a truncated-cone. For the HE particularization in the RRAM CMA some further assumptions are employed for the sake of simplicity:

(a) Constant thermal conductivity, i.e., *k_th_*(*x*,*T*) = *k_th_*. Neither geometric nor temperature dependencies are considered. In most cases, the CF thermal conductivity is the one considered.
(b) A single temperature in the whole conductive filament [38,78] is taken into account (this means a strong simplifying approach). Some models for circuit simulation can account for two different temperatures [79], this is a good strategy since the key (higher) temperature at the CF narrowing, where the CF is ruptured, is decoupled from the main CF bulk temperature; this latter temperature does not increase in the same manner. See Figure 4c in [80], where the CF temperature along the dielectric is plotted for different voltages. It is clear that the temperature is much higher in the CF narrowing while it shows a different behavior for the main CF body. The model with two different CF temperatures is more complex, hence, this issue has to be taken into account when dealing with circuits including hundreds or thousands of components.

**Figure 1:**
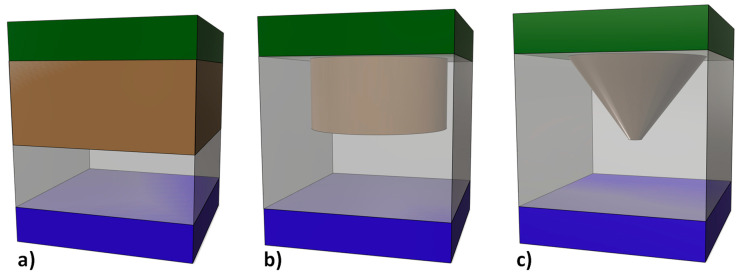
Different conductive filament shapes employed in RRAM compact models for circuit simulation. We will use this CF shapes in the thermal models described below. (**a**) CF that occupies all the modeling domain, (**b**) cylindrical CF, (**c**) truncated-cone shaped CF.

Different RRAM cell schematics are shown in Figure 1. Assuming filamentary conduction, the CF evolution has to be calculated to describe RS operation and determine the device current. Several CF types (shapes) from the CMA employed in the literature are represented in Figure 1.

### 2.2. A Numerical Approach for the Heat Equation

Equation (1) is essential to all types of RRAM physical simulators. It is usually auto-consistently solved with other differential equations (Poisson equation, kinetic equations for the chemical reactions that control the CF evolution, etc.) [21,23,34,53,70,77,81]. Since the most common device structure is not based on curved surfaces or volumes, even in the more scaled cases, a finite difference approach could be a reasonable choice that simplifies the grid and the differential equations discretization. Boundary conditions are key to describe correctly the physics of the device operation. In many simplified models, the electrodes are supposed to be perfect heat sinks and, therefore, Dirichlet’s boundary conditions are established at the electrode-dielectric interface (in most cases room temperature is assumed at this interface). However, to correctly describe heat transfer between the conductive filament, the dielectric layer and the electrodes, some parts of the latter should be included in the simulation domain (SD). The SD lateral interfaces (perpendicular to the dielectric-electrode interface) are usually described by Neumann boundary conditions. Sometimes the temperature derivative in the normal direction of these interfaces is assumed to be null. This means adiabatic conditions accordingly with Fourier’s law for heat conduction.

**Figure 2:**
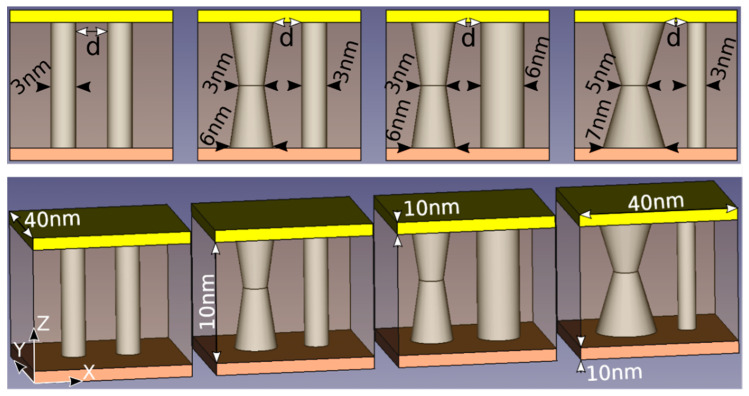
Simulated RRAMs. Four different LRS situations are considered, in all cases a double conductive filament was employed assuming a distance, d, between them (the bottom and top figures are the same in each case from different perspectives). The bottom electrode is assumed to be Si-n^+^ and the top electrode is made of Ni, the dielectric consists of HfO_2_. The conductive filament shapes employed are shown for the different cases under study, they are assumed to be metallic-like, formed by Ni atom clusters [24,55,80]. The physical parameters are the same employed in the simulation in [29].

We have included here some results to illustrate the HE role in a RRAM simulator. Our simulator calculates the RRAM current in the LRS. The internal resistance calculation is performed assuming fully formed metallic-like conductive filaments of different shapes. In addition to the current calculation, we solve the 3D HE [29]. In the SD we have included the dielectric stack and part of the electrodes; precisely, 10 nm of the Si-n^+^ (bottom electrode) and Ni (top electrode). Consequently, the SD thermal boundary is shifted from the dielectric/electrode interfaces to the electrodes, as commented above. A 40 nm (*X* axis) × 40 nm (*Y* axis) × 30 nm (Z axis, vertical axis running from the Si layer to the Ni layer) SD is considered, see Figure 2.

Dirichlet’s boundary conditions were supposed at the outer electrode layer surfaces. The constant temperature located outside the device was room temperature (a reasonable assumption accounting for the high electrode thermal conductivity). For the SD lateral faces we employed perfectly matched layers (PML) [82], an improved implementation of Neumann boundary conditions since PML are particularly appropriate for differential equation solution to deal with open boundary problems, our case here [83]. Joule heating takes place in the CFs. Some devices (schematics shown in Figure 2) were simulated. In our simulations we included two CFs to account for a different device configuration with respect to conventional studies with just one CF.

**Figure 3:**
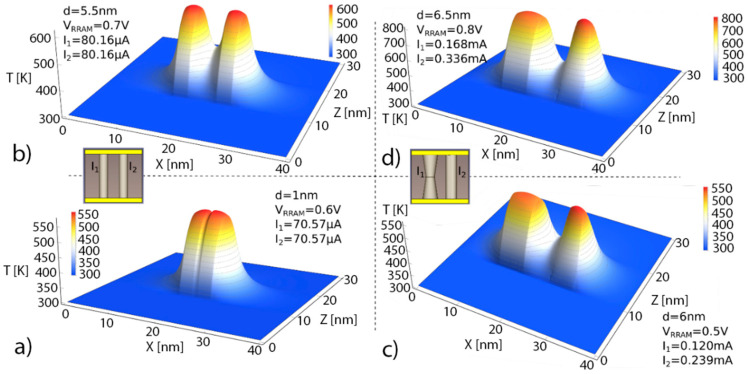
Temperature plots for some of the devices simulated as explained above (see the insets), the cross-section cuts corresponds to y = 20 nm in our SD. (**a**) RRAM with two cylindrical CFs (diameter = 3 nm) separated 1 nm apart for a bias of 0.6 V. (**b**) RRAM with two cylindrical CFs (diameter = 3 nm) separated 5.5 nm apart for a bias of 0.7 V. (**c**) RRAM with one cylindrical CF (diameter = 6 nm) and a symmetrical truncated-cone shaped CF (low diameter = 3 nm, high diameter = 6 nm) separated 6 nm apart for a bias of 0.5 V. (**d**) RRAM with one cylindrical CF (diameter = 6 nm) and a symmetrical truncated-cone shaped CF (low diameter = 3 nm, high diameter = 6 nm) separated 6.5 nm apart for a bias of 0.8 V. For the sake of visibility, some of the 3D plots are rotated with respect to the 2D CF scheme.

In Figure 3, symmetric temperature distributions are observed in (a) and (b), corresponding to similar CFs, and therefore, to alike Joule heating effects. See the effects of the low thermal conductivity in the dielectric. According to Fourier’s law for heat conduction, the heat flux, q, is equal to the product of thermal conductivity, k_th_, and the negative local temperature gradient ($q=-k_{th}\nabla T$). Since the HfO_2_ thermal conductivity is around 1 W/(K m), the temperature drops off rapidly around the CF. See also the effects of the dielectric-electrode boundary at z = 10 nm and z = 30 nm, the temperature reduction is different from what is seen in the CF perpendicular direction along the *x*-axis. The maximum temperature is obtained in the narrowest section for the symmetrical truncated-cone shaped CF, as seen in (c) and (d). At this point, the physical mechanisms behind RS are more active and, consequently, they trigger the CF rupture at this location. See the thermal connection between the CFs for the different shapes and distances in between; in this respect, in tree-branch shaped filaments, the destruction of the branches and the thermal and current redistribution in the remaining intertwined branches makes the reset a complicated process.

**Figure 4:**
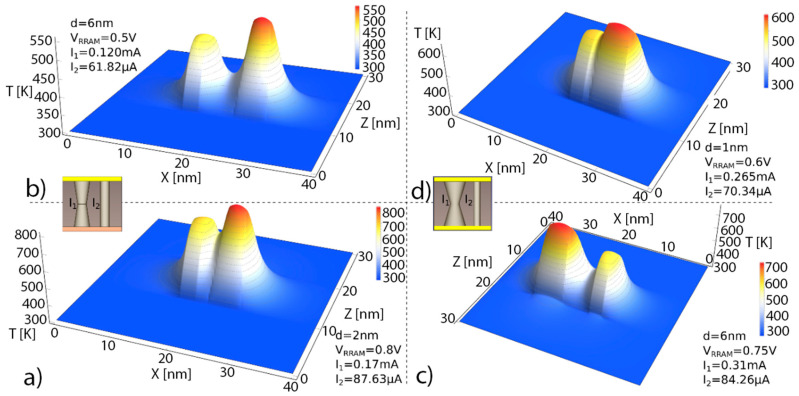
Temperature plots for some of the devices simulated as explained above (see the insets), the cross-section cuts corresponds to y = 20 nm in our SD. (**a**) RRAM with one cylindrical CF (diameter = 3 nm) and a symmetrical truncated-cone shaped CF (low diameter = 3 nm, high diameter = 6 nm) separated 2 nm apart for a bias of 0.8 V. (**b**) RRAM with one cylindrical CF (diameter = 3 nm) and a symmetrical truncated-cone shaped CF (low diameter = 3 nm, high diameter = 6 nm) separated 6 nm apart for a bias of 0.5 V. (**c**) RRAM with one cylindrical CF (diameter = 3 nm) and a symmetrical truncated-cone shaped CF (low diameter = 5 nm, high diameter = 7 nm) separated 6 nm apart for a bias of 0.75 V. (**d**) RRAM with one cylindrical CF (diameter = 3 nm) and a symmetrical truncated-cone shaped CF (low diameter = 5 nm, high diameter = 7 nm) separated 1 nm apart for a bias of 0.6 V. For the sake of visibility, some of the 3D plots are rotated with respect to the 2D CF scheme.

In Figure 4 simulations of other RRAM configurations are shown. See that small changes in the device voltage can lead to important temperature variations in the CF bottleneck where RS mechanisms are thermally enhanced. In (c) and (d), the thermal cross-talk between CFs is observed when the distance between CFs is around 1 nm. For much higher inter CF distances, a lower thermal connection results even for higher currents.

### 2.3. Explicit Heat Equation Solutions

#### 2.3.1. RRAM with a Cylindrical Filament (Steady-State Operation, No Heat Transfer Term)

**Figure 5:**
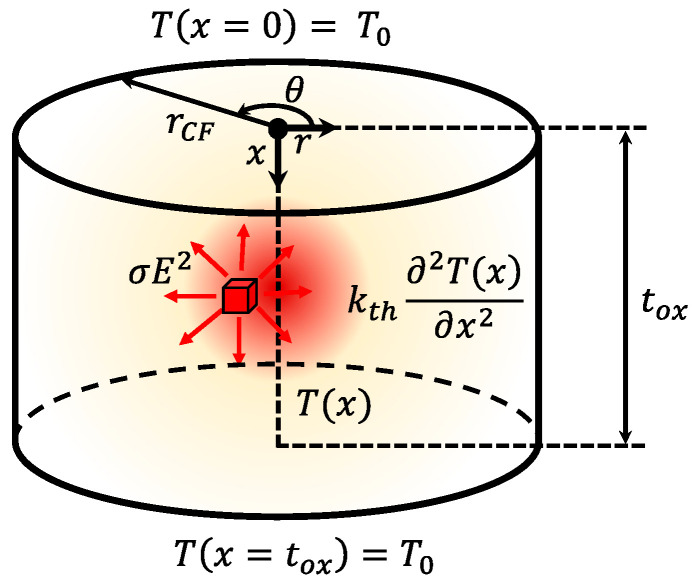
Schematic showing the different elements considered to solve the simplified HE in a cylindrical and homogenous conductive filament.

In the case under consideration, we assume a cylindrical CF with constant electrical and thermal conductivities. The boundary conditions are established at the extremes of the filament (*x* = 0 and *x = t_ox_*, respectively, with temperatures *T*(*x* = 0) = *T*(*x* = *t_ox_*) = *T*_0_, where t_ox_ stands for the dielectric thickness), see Figure 5.

From Equation (2), we obtain [38]:

(3) $$\sigma E^{2}=-k_{th}\frac{\partial^{2}T(x)}{\partial x^{2}}$$

where *E* is the constant electric field in the CF (*E = V_RRAM_/t_ox_*). Figure 5 shows the different elements taken into account to solve the HE. The solution for the maximum temperature in the middle of the CF, with the boundary conditions sketched in Figure 5, is:

(4) $$T_{max}=T_{0}+\frac{\sigma \;t_{ox}^{2}\;E^{2}}{8\;k_{th}}$$

The single temperature that will represent the whole CF thermal state is chosen to be *T_max_*, as shown below (see Appendix D.3 in [84]),

(5) $$T_{max}=T_{0}+\frac{\sigma \;V_{RRAM}^{2}}{8\;k_{th}}$$

Henceforth, this will be our thermal model 1, TM1. This assumption is justified by the fact that this maximum value controls the physical mechanisms that lead to the CF narrowing in a reset process and finally to the CF rupture. Nevertheless, among other issues in this model, the calculation of the ohmic resistance as the CF heats up is not correctly solved since the main CF body remains at a temperature much lower than the hottest spot where the maximum temperature is achieved. The Verilog-A implementation is shown in Table 1 (TM1).

#### 2.3.2. RRAM with a Cylindrical Filament Including a Heat Transfer Term (Steady-State Operation)

**Figure 6:**
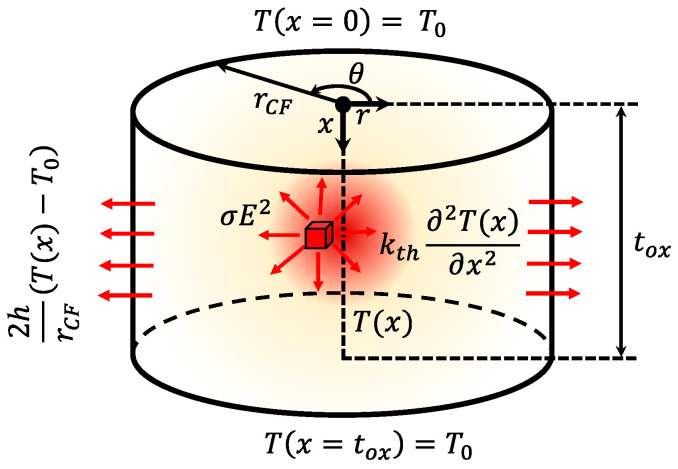
Sketch of a cylindrical filament with the different terms in the HE shown in Equation (6), including the heat transfer term.

We have added a term to account for the heat transfer from the CF to the surrounding insulator to Equation (3), see Figure 6. The heat losses are included by means of the heat transfer coefficient (h) [31,38,79,85], see Equation (6):

(6) $$\sigma \;E^{2}=-k_{th}\frac{\partial^{2}T\left(x\right)}{\partial x^{2}}+2\;h\frac{T\left(x\right)-T_{0}}{r_{CF}}$$

where *r_CF_* stands for the conductive filament radius.

Equation (6) can be solved and the result is given below [27],

(7) $$T_{max}=T_{0}+\frac{\sigma \;E^{2}\;r_{CF}\;{\left(e^{\alpha }-1\right)}^{2}}{2\;h\;\left(e^{2\alpha }+1\right)}$$

where:

(8) $$\alpha =\frac{t_{ox}}{2}\sqrt{\frac{2\;h}{k_{th}\;r_{CF}}}$$

In this case, as before, the electric field is assumed constant, and the CF temperature is considered to be *T_max_*, as calculated in Equation (7). We will consider this the thermal model 2, TM2 (see the Verilog-A code in Table 1).

#### 2.3.3. RRAM with a Truncated-Cone Shaped Filament Including Heat Transfer Coefficient (Steady-State Operation)

**Figure 7:**
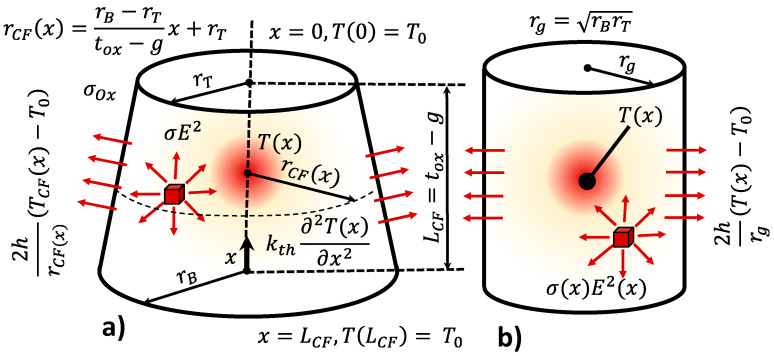
(**a**) Energy dissipation terms included in the heat equation and geometrical domain for the CF thermal description, (**b**) cylindrical CF equivalent employed to simplify the HE solution and obtain a compact analytical expression for the CF temperature. Note that the conductive filament length (*L_CF_*) can be lower than the oxide layer (*t_ox_*), due to the gap (*g*) between the top electrode and the conductive filament tip (see [27,78,86,87,88]).

The boundary conditions and the CF geometry for this model are shown in Figure 7. Notice that the CF radius $r_{CF}(x$) of the truncated cone depends on the x position. Consequently, the heat equation can be expressed now as follows:

(9) $$\sigma \left(x\right)\;E{\left(x\right)}^{2}-\frac{2h}{r_{CF}\left(x\right)}\left(T\left(x\right)-T_{0}\right)=-k_{th}\frac{\partial^{2}T\left(x\right)}{\partial x^{2}}$$

This equation cannot be solved analytically because of the variable CF radius, $r_{CF}(x$). In order to obtain an approximated analytical solution, we considered a transformation to simplify. A truncated-cone shaped CF with constant conductivity was found to be approximately equivalent in terms of this calculation to a cylinder with radius ($r_{g}=\sqrt{r_{T}r_{B}}$) and variable conductivity $\sigma_{CF}(x$) [27], see Figure 7. For a fixed applied voltage, we include the maximum electric field. This value is affected by the ratio between the two truncated-cone radii ($\eta =r_{T}/r_{B}$). Under this assumption, the following simplified HE was obtained:

(10) $$\frac{\sigma \;V_{RRAM}^{2}}{L_{CF}^{2}\;\eta }-\frac{2h}{r_{g}}\left(T\left(x\right)-T_{0}\right)=-k_{th}\frac{\partial^{2}T\left(x\right)}{\partial x^{2}}$$

Using this parameter:

(11) $$\alpha =L_{CF}\sqrt{\frac{2h}{k_{th}\;r_{g}}}$$

The value of *T_max_* can be obtained as follows (the one we assume for the whole CF, this will be the thermal model 3, TM3, see the Verilog-A code in Table 1),

(12) $$T_{max}=T_{0}+\frac{r_{g}\;\sigma \;E^{2}}{\eta \;h}\left[\frac{1}{2}-\frac{e^{\frac{\alpha }{2}}}{e^{\alpha }+1}\right]$$

#### 2.3.4. RRAM with a Truncated-Cone Shaped Filament Including Heat Transfer Coefficient (Steady-State Operation) and Two Temperature Values to Represent the CF Thermal Behavior

The use of two different CF temperatures allows a more accurate thermal description of the CF narrowing, where the temperature rises due to the thermal run-away process that leads to the RS operation, and a CF bulk temperature that accounts for the temperature in the CF wider part. This CF region could be left almost untouched in the sequence of set and reset processes. We will consider this version the thermal model 4, TM4 (see in Table 1 a Verilog-A implementation of the first thermal models proposed here). In the approach we followed, the electric field is calculated by means of two components associated with the CF top and bottom parts [79]. In this respect, the electric field can be calculated as: $E_{\left(T,B\right)}=\frac{V_{\left(T,B\right)}}{L/2}$, where $V_{\left(T,B\right)}$ is obtained considering the voltage divider formed by the resistances associated with the CF top and bottom portions (see the Appendix in [79]).

**Figure 8:**
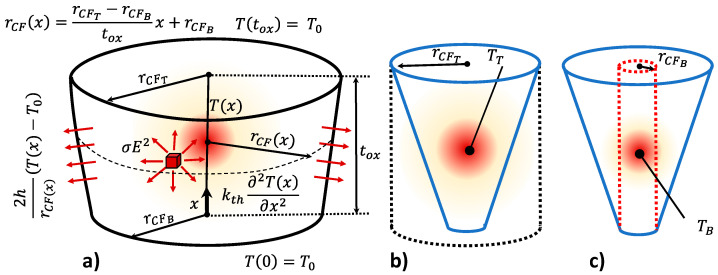
RRAM cell CF scheme for the thermal model based on two different CF temperatures. (**a**) Original filament with the corresponding boundary conditions. Cylindrical CFs (shown in dashed lines) employed to compute the (**b**) top temperature *T_T_* and (**c**) the bottom *T_B_*.

In the approximation strategy developed here, the maximum temperature (see the previous thermal models) is obtained for two cylinders of different radii (associated to the top and bottom CF temperatures), see Figure 8. These values are assumed to be the temperatures at the main CF volumes linked to the cylinders: for the thicker CF section (Figure 8b), $T_{T}$, and for the narrow CF (Figure 8c), $T_{B}$. The analytical expression for the temperature calculation is given below:

(13) $$T_{\left(T,B\right)}=T_{0}+\frac{\sigma_{\left(T,B\right)}r_{CF_{\left(T,B\right)}}E_{\left(T,B\right)}^{2}}{2h}\left(1-cosh\left(\frac{\alpha_{\left(T,B\right)}t_{ox}}{2}\right)\right)+\frac{dT_{0_{\left(T,B\right)}}}{\alpha_{\left(T,B\right)}}sinh\left(\frac{\alpha_{\left(T,B\right)}t_{ox}}{2}\right)$$

where, parameters $\alpha_{\left(T,B\right)}$ and $dT_{0_{\left(T,B\right)}}$ are given in the equations below [79]:

(14) $$\alpha_{\left(T,B\right)}=\sqrt{\frac{2\;h}{k_{th}r_{CF_{\left(T,B\right)}}}}$$

(15) $$dT_{0_{\left(T,B\right)}}=\frac{\sigma_{\left(T,B\right)}r_{CF_{\left(T,B\right)}}E_{\left(T,B\right)}^{2}tanh\left(\frac{\alpha_{\left(T,B\right)}t_{ox}}{2}\right)}{\sqrt{2\;k_{th}\;h\;r_{CF_{\left(T,B\right)}}}}$$

Equation (16) includes the CF conductance temperature dependence, assuming a metallic behavior:

(16) $$\sigma_{\left(T,B\right)}=\frac{\sigma_{0}}{1+\alpha_{T}\left(T_{\left(T,B\right)}-T_{0}\right)}$$

where $\sigma_{0}$ stands for the CF conductivity at room temperature ($T_{0}$) and $\alpha_{T}$ is the conductivity temperature coefficient. The Verilog-A code is shown in Table 1 (TM4).

### 2.4. Energy Balance in the Device

If we apply the first law of thermodynamics in terms of an energy balance in the device active region, we would obtain Equation (17) [78]:

(17) $${\dot{e}}_{generated}-\kappa \left(T\left(t\right)-T_{0}\right)=C_{th}\frac{\partial T\left(t\right)}{\partial t}$$

where *T_0_* stands for the temperature of the dielectric that surrounds the CF (usually assumed at room temperature) and κ is the inverse of the thermal resistance. In this case we do not account for a temperature distribution along the CF as in Equation (2). Our perspective here accounts for the whole CF, and even its surroundings, which is represented by a single temperature. In addition, we do not follow, as above, a scheme based on the HE solution and the association of the maximum temperature with the CF. The power generated can be calculated as *V_RRAM_(t)I_RRAM_(t)*, although we will employ *V(t)I(t)* for short. We can study this differential equation accounting for different operation regimes.

#### 2.4.1. Steady-State

This regime works well under the conventional ramped voltage stress, RVS. With long ramps to switch the device, Equation (17) can be written as follows:

(18) $${\dot{e}}_{generated}=\kappa \left(T\left(t\right)-T_{0}\right)$$

The power generated in the conductive filament (CF) (Joule heating effects calculated as current x voltage) equals the power dissipated toward the electrodes and the dielectric. Under the consideration of a single temperature to characterize the device active region, assuming that the electrodes and the dielectric are perfect heat sinks at a fixed temperature, *T_0_*, Equation (18) can be written as follows:

(19) $$R_{th}=\frac{T-T_{0}}{V\;I}$$

where *R_th_* stands for the effective thermal resistance (it depends on the device physical features and is associated with heat conduction [89]). Using this simple model (thermal model 5, TM5), the device temperature can be estimated from Equation (19) [88,90,91,92,93,94].

**Figure 9:**
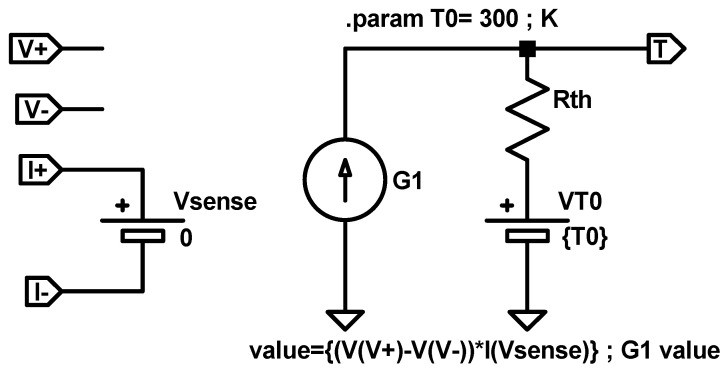
Equivalent electric circuit for the RS device thermal model based on a thermal resistance *R_th_*.

At circuit simulation level, Equation (19) can be implemented with the equivalent electrical sub-circuit shown in Figure 9. The heat dissipated power is represented by a dependent current source whose value is described as $G_{1}=VI$, where the voltage is determined by the two input sub-circuit terminals $V^{+}$ and $V^{-}$, and the current is sensed by a null voltage source $V_{sense}$ connected in series between the input terminals $I^{+}$ and $I^{-}$. The thermal resistance is represented by an electrical resistance, $R_{th}$, and the room temperature by a constant voltage source, $T_{0}$, with a value that equals the room temperature ($T_{0}$), in K. The output sub-circuit terminal (T) provides a voltage that represents the device temperature T (in K).

#### 2.4.2. Non-Steady-State Approach

Models based only on thermal resistances do not provide capacitive effects in terms of transient operation (mostly related to pulsed voltage stress, PVS, that can be employed to tune the device resistance in a multilevel operation regime, as needed in neuromorphic circuits). In order to include thermal inertia in the model, a capacitor (in the electrically equivalent thermal circuit) is added, *C_th_*, the thermal or heat capacitance. This approach is used in different RRAMs compact models (thermal model 6, TM6), [78,95,96]. We can reformulate Equation (17) to the following expression:

(20) $$VI=\frac{T-T_{0}}{R_{th}}+C_{th}\frac{dT}{dt}$$

From Equation (20), the temperature can be obtained as:

(21) $$T=T_{0}+V\;I\;R_{th}-\tau_{th}\frac{dT}{dt}$$

where $\tau_{th}$ (thermal time constant) is defined as follows:

(22) $$\tau_{th}=C_{th}\;R_{th}$$

Equation (20) can be solved analytically if we assume a constant voltage (see Equation (23)).

(23) $$T\left(t\right)=T_{0}+V\;I\;R_{th}\left(1-e^{-\frac{t}{\tau_{th}}}\right)$$

If we apply a time-dependent voltage, *V*(*t*), Equation (23) does not work. In this case, we have to solve numerically Equation (20). Assuming a new function, *X(t) = T(t) − T_0_*, we obtain,

(24) $$V\left(t\right)I\left(t\right)=\frac{X\left(t\right)}{R_{th}}+C_{th}\frac{dX\left(t\right)}{dt}$$

Discretizing under equally distant temporal points *t_i+_*_1_ *= t_i_* + Δ*t*:

(25) $$V_{i}I_{i}=\frac{X_{i}}{R_{th}}+C_{th}\frac{X_{i+1}-X_{i}}{\Delta t}$$

which gives us:

(26) $$R_{th}V_{i}I_{i}=X_{i}+\tau_{th}\frac{X_{i+1}-X_{i}}{\Delta t}$$

and consequently,

(27) $$X_{i+1}=\frac{\Delta t}{\tau_{th}}\left(R_{th}V_{i}I_{i}-X_{i}\right)+X_{i}$$

**Figure 10:**
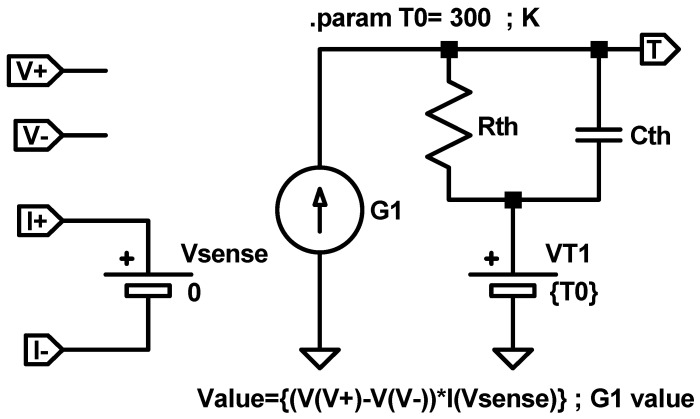
Equivalent electric circuit of a RRAM thermal model based on a thermal resistance and capacitance to implement Equation (20).

Finally, the device temperature could be calculated as *T_i+1_ = X_i+1_ + T_0_*. From a circuit simulation point of view, Equation (20) can be implemented with the equivalent electrical sub-circuit shown in Figure 10. The values of the different electric elements and the role of the pins is the same as in Figure 9. However, a capacitor has been added to account for the thermal capacitance.

The values κ = 2.8–25 µJ/K s (*R_th_* = 4 × 10^4^–3.5 × 10^5^ K/W) and *C_th_* = 0.04–1.1 pJ/K were employed in [78]. In [95], the following values were given: *C_th_* = 0.318 fJ/K and *τ_th_* = 0.23 ns, from them the thermal resistance can be extracted *R_th_ = τ_th_/C_th_* = 7.23 × 10^5^ K/W. The thermal resistance in [91] was taken *R_th_* = 5 × 10^5^ K/W. An estimation of 33 ps for the thermal time constant is reported in [18], which has to be compared to the electric pulse-width to assess the importance of thermal transient effects in conventional RRAM operation. The heat capacitance can be calculated as *C_th_ = C_p_ t_ox_ A* [18], where A is the CF effective area, the effective CF length that approximately corresponds to the dielectric thickness (*t_ox_*) and *C_p_* is the volumetric heat capacity (calculated as *ρ* × *c*, Equation (2)) [89]. As detailed in [18], the thermal resistance can be calculated as follows:

(28) $$R_{th}\approx \frac{t_{ox}}{k_{th}\;A}$$

Assuming as the reference material Hf, we have the following values *k_th_* = 23 Wm^−1^K^−1^, and *C_p_* = 1.92 JK^−1^cm^−3^. Therefore, the thermal time constant can be estimated as:

(29) $$R_{th}C_{th}=\frac{C_{p}\;t_{ox}^{2}}{k_{th}}$$

which is 33 ps for the case considered (*t_ox_* = 20 nm).

A quick estimation to assess the role of the thermal resistance (*R_th_* = 4 × 10^4^ K/W) can be performed if we assume an ideal device in the LRS under a steady-state regime with *I_RRAM_* = 1 mA and *V_RRAM_* = 1 V; using Equation (19), we would have *T* = *T_0_* + *R_th_* × *I* × *V* = *T_0_* + 40 K, or *T* = *T_0_* + 500 K, if *R_th_* = 5 × 10^5^ K/W.

**Figure 11:**
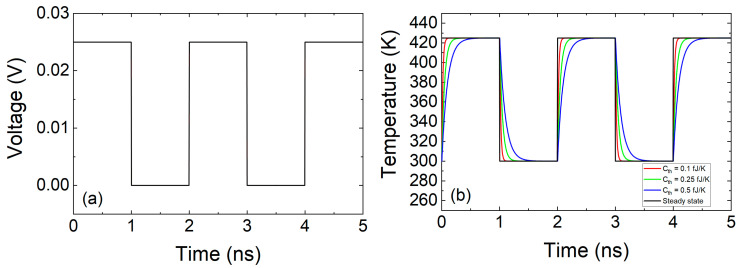
(**a**) Voltage applied to the device versus time, (**b**) Temperature versus time obtained for different values of *C_th_* (assuming *R_th_* = 2 × 10^5^ K/W).

The role of the heat capacitance can be easily seen in Figure 11. For a pulsed voltage signal of amplitude (peak to peak)= 0.025 V, offset = 0.0125 V and f = 0.5 GHz applied in an ideal device whose resistance is assumed to be *R_RRAM_* = 1 Ω, the temperature can be obtained from Equation (27) for *R_th_* = 2 × 10^5^ K/W and *C_th_* = 0.1 fJ/K (*τ_th_* = 20 ps), *C_th_* = 0.25 fJ/K (*τ_th_* = 50 ps), *C_th_* = 0.5 fJ/K (*τ_th_* = 100 ps).

As can be seen in the previous figure, the device thermal response should be faster than the electric pulses employed for an operation free of delays (“inertia”) due to thermal effects. In fact, experimental techniques have been developed to estimate the device temperature with the application of ultra-short electrical pulses to RRAMs [97,98].

We have made use of some of the thermal models reported above. They cannot exist on their own since the conductive filament kinetics need to be considered to describe the device RS operation and, in doing so, calculate the current. The use of the thermal models TM1-TM4 has been presented previously in [27,56,79]. We show here some results of the Stanford model [78,88,93,95] along with the thermal models TM5 and TM6. For the device description, taking into consideration that no experimental data fitting is considered (since it was performed in the references where the models were introduced), we assumed a set of model parameters close to the one suggested in [88], see Table 2.

**Figure 12:**
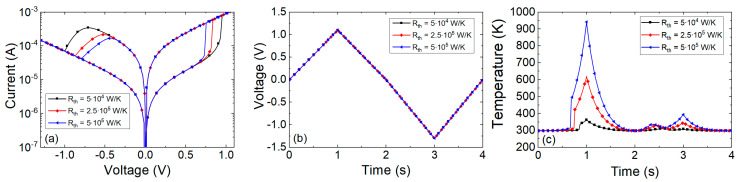
Simulations performed making use of the Stanford model including the TM5 with different thermal resistances. (**a**) Current versus voltage applied to the device, (**b**) voltage signal applied to the device, (**c**) temperature versus time.

Several I-V curves were plotted in Figure 12 considering different thermal resistances. As can be seen, the main dissimilarities are found in the set and reset regions. For the Stanford model, the highest temperatures are found in the set process, see that the Joule heating is higher in the set process and in the interval of positive voltages above V_SET_. This is coherent with experimental findings that show that the Joule heating role is essential to describe the SET kinetics [81,99]. On average, these maximum temperatures achieved are in line with the estimations performed above for the thermal resistances considered. It is important to highlight the importance of the thermal resistance in the device design. See that higher *R_th_* values lead to lower set and reset voltages to produce RS, and hence, lower power consumption. From this viewpoint, higher thermal resistances (i.e., devices showing a character thermodynamically more adiabatic with respect to the surroundings) might be more interesting. Since the heat flux from the CF to the metallic electrodes (operating these as heat sinks, a reasonable assumption, allows to calculate the heat flux with the material 3D thermal conductivity) could be in the same order of magnitude for different RRAMs, the dielectric could be the key (save the role of the contact thermal resistance). In this respect, we have to call the reader’s attention to the fact that the usual RRAM dielectrics are grown in nanometric layers; consequently, the real thermal conductivity due to phonon quantization is far from the values corresponding to the corresponding 3D dielectric materials, as it was shown in [100,101,102].

**Figure 13:**
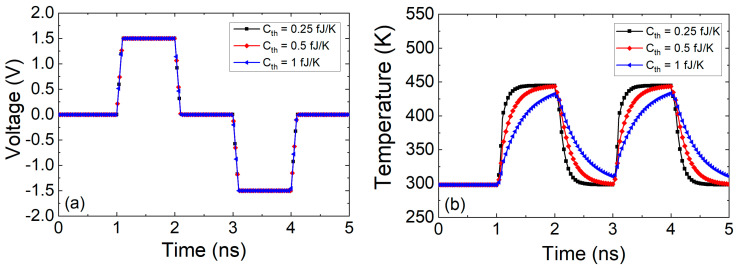
Simulations performed with the Stanford model (including TM6) making use of different thermal capacitances, *C_th_*, assuming a common value of the thermal resistance, *R_th_* = 4 × 10^5^ K/W. (**a**) Voltage applied to the device versus time, (**b**) Temperature versus time.

In the case of TM6 along with the Stanford model (using Table 2), we have plotted the results of a simulation using a pulsed voltage signal in Figure 13. A voltage signal such as the one in Figure 12b is close to DC, therefore the use of TM6 instead of TM5 is irrelevant. However, if a fast voltage signal is employed (Figure 13a), a high enough thermal capacitance makes a difference in terms of the device temperature transient (see Figure 13b).

The thermal time constants (Equation (22)) produced obvious delays for a voltage signal such as the one in Figure 13. These delays could seriously affect the device operation under pulsed input signals since RS is most of the times linked to thermal effects [1,24,53,81,99]. The thermal time constants corresponding to Figure 13b are the following: 0.4, 0.2 and 0.1 ns. These values are large compared to the value (33 ps) given by Ielmini [18] (we do not go into details of device materials at this point). An estimation of the *C_th_* associated to a CF in a conventional RRAM, as described in [18], could help to shed light on this issue. Let us assume a cylindrical CF radius of 5 nm in a dielectric layer of 20 nm thick, if the heat capacity of Hf is considered (*C_p_* = 1.92 JK^−1^cm^−3^) the CF thermal capacitance would be (*C_th_* = *C_p_ t_ox_ A*) 0.003 fJ/K. Therefore, a value *τ_th_* = 1.2 ps is expected for the same thermal resistance employed in Figure 13. Although this thermal time constant is short, different authors [78,95] have used thermal capacitances values that lead to devices with higher thermal constants. A thermal device model described by Equation (20) and the thermal capacitance of an average CF produces so low thermal time constants that no transient term in Equation (20) would be worth being taken into account. From the experimental viewpoint, no delays linked to thermal inertia would be seen for conventional memory pulsed signals. Nevertheless, current transients on longer time scales than the previously calculated *τ_th_*, linked to some extent to thermal effects have been reported previously [78]. In this respect, the thermal model, i.e., Equation (20), might not be enough to accurately describe RRAM thermal response.

The coupling of the CF temperature to a heat sink at room temperature with just two parameters (thermal resistance and capacitance) could not be described by the thermal features of a nanometric CF. We could use an intermediate temperature corresponding to an average region surrounding the CF that could help to build two different thermal circuits, one accounting for the coupling between the CF and this intermediate surrounding region (it could include the dielectric and electrode zones closer to the CF), and a second one coupling between this intermediate region and the outside world. This is in line with previous thermal descriptions (although introduced in another context) employed for magnetic current sensors [103] and AlGaAs/GaAs HBTs [104]. For this purpose, we present the following model. We will show below that the model based on Equation (20) can be used if a thermal capacitance that accounts for the whole device is taken into consideration. This means using a parameter much higher than the exclusively associated to the CF. In doing so, as always in compact modeling, the simplicity and accuracy trade-off has to be considered.

#### 2.4.3. Non-Steady-State Approach with Two Different Temperatures Associated to the Device (Second-Order Memristor)

**Figure 14:**
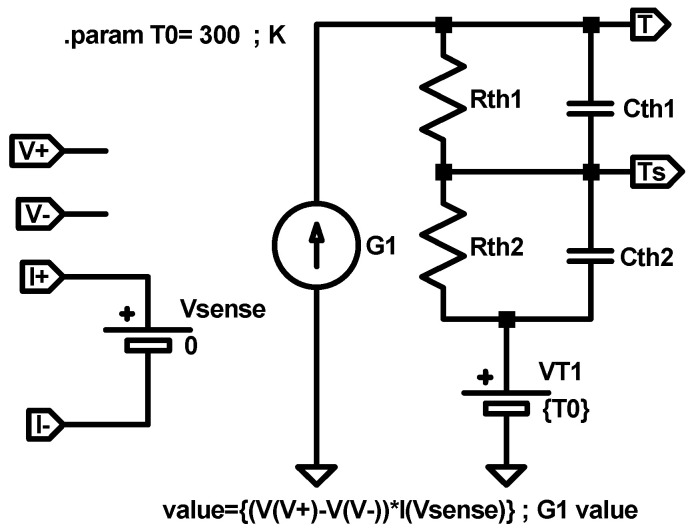
Equivalent electric circuit of a RRAM thermal model based on a double thermal circuit described by Equations (30) and (31).

This thermal model, as suggested above, employs two different temperatures to describe the device from an energy balance perspective: the internal device temperature (approximately the CF temperature, T) that affects the RS mechanisms linked to the CF creation and destruction and a second temperature, associated to the CF surrounding regions (an effective temperature, *T_S_*). The latter influences the internal device temperature T but it shows a different time evolution. The device intermediate surrounding region (at temperature *T_S_*) is characterized by an outer boundary assumed to be at room temperature (*T_0_* = 300 K). This outer boundary is considered to be far away from the RS active region. The intermediate surrounding region can include different material layers; therefore, effective thermal constants are employed to account for the heat flux between this region and the exterior zone. Besides, the coupling between the inner (CF volume) and the intermediate CF surrounding region could be modeled by an effective thermal resistance and thermal capacitance: *R_th1_* and *C_th1_*. Under this approach, the device can be described by the following two equations (we assume this procedure to be the thermal model 7, TM7; see the circuital implementation in Figure 14).

(30) $$C_{th1}\frac{d\left(T-T_{S}\right)}{dt}=V\left(t\right)I\left(t\right)-\frac{1}{R_{th1}}\left(T-T_{s}\right)$$

(31) $$C_{th2}\frac{dT_{s}}{dt}=V\left(t\right)I\left(t\right)-\frac{1}{R_{th2}}\left(T_{s}-T_{0}\right)$$

where *V*(*t*)*I*(*t*) allows the determination of ${\dot{e}}_{generated}$. The power dissipated by Joule heating affects the intermediate surrounding region temperature (modeled with parameters *R_th2_* and *C_th2_*) that accounts for the coupling between this region and the thermalized device exterior region. The approach described here is in line with the description of a second order memristor [100].

**Figure 15:**
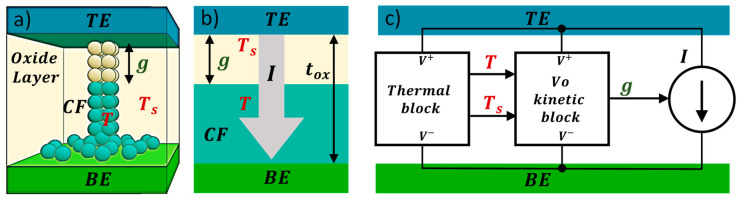
(**a**) Three-dimensional view of the Stanford scheme to model the different device areas (TE: top electrode, Oxide Layer, CF: conductive filament and BE: bottom electrode), (**b**) model parameters, g: gap between the TE and the filament tip and *t_ox_*: dielectric thickness, (**c**) subcircuit representation for the implemented model. The connection between blocks represents the states variables used: g, which depends on kinetic block and it is linked to the two temperatures (*T*, *T_S_*).

The simulation was performed with this thermal model integrated in the Stanford compact model that was employed previously, see Figure 15.

**Figure 16:**
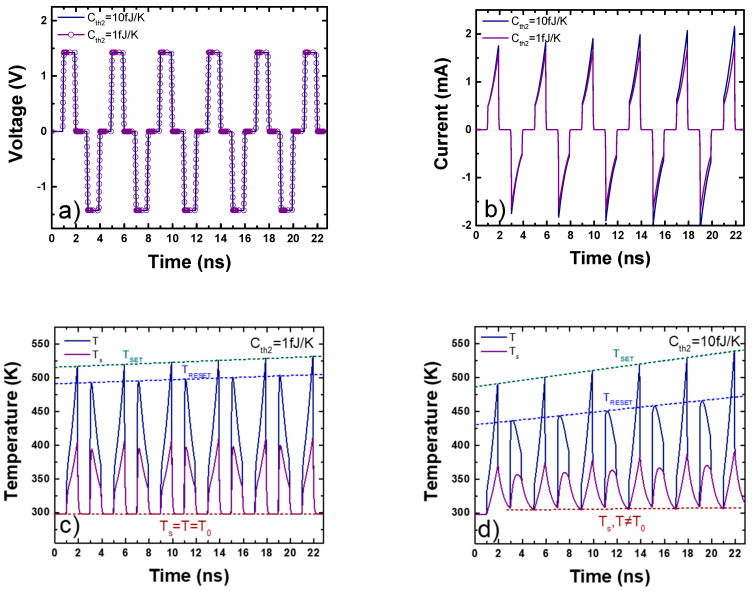
RRAM simulation making use of the Stanford model including a double RC thermal model *R_th1_* = 40 kK/W, *R_th2_* = 40 kK/W, *C_th1_* = 0.003 fJ/K and C_th2_ with values 1 fJ/K and 10 fJ/K. (**a**) Applied voltage pulses for consecutive set and reset, (**b**) temporal current evolution, (**c**) temporal evolution of device filament temperature (*T*) and the intermediate surrounding region (*T_S_*) with *C_th2_* = 1 fJ/K and (**d**) *C_th2_* = 10 fJ/K.

The results obtained for the set of parameters of the standard Stanford parameters [88] and the thermal model shown in Figure 14; Figure 15 are given in Figure 16.

Depending on *C_th2_*, different CF and intermediate surrounding region temperatures transient responses are obtained, producing the corresponding effects on the device current. This is noticeable when a consecutive series of set and reset pulses are applied, as shown in Figure 16a, in which a sequence of set pulses (1.5 V with 1 ns on time and 0.1 ns rise and fall times) and reset pulses (−1.5 V with 1 ns on time and 0.1 ns rise and fall times). In the first configuration, with the thermal capacities *C_th1_* = 0.003 fJ/K, *C_th2_* = 1 fJ/K and thermal resistances *R_th1_* = *R_th2_* = 40 kK/W, the current evolution is shown in Figure 16b. Figure 16c shows the CF (T) and intermediate surrounding region (*T_S_*) evolution. In this first configuration, the corresponding transient shows low thermal inertia; after the pulse application, both temperatures reach room temperature (*T* = *T_S_* = *T_0_*). The devices show a slight increase in the maximum temperatures obtained in the set (*T_SET_*) and reset (*T_RESET_*) processes (Figure 16c).

In the second configuration, thermal capacities *C_th1_* = 0.003 fJ/K, *C_th2_* = 10 fJ/K and thermal resistances *R_th1_* = *R_th2_* = 40 kK/W, the current evolution is plotted in Figure 16b. Figure 16d shows the CF (T) and intermediate surrounding region (*T_S_*) evolution. In this second configuration, the thermal inertia is higher in the second thermal circuit, after each set/reset pulse, the temperatures cannot go back to room temperature (*T*, *T_S_* ≠ *T_0_*). As a result, each new cycle starts from a higher temperature than in the previous cycle; therefore, the maximum set (*T_SET_*) and reset (*T_RESET_*) temperatures show a growing trend (see Figure 16d). This temperature increase over the cycles implies that the device CF gap decreases in each new cycle, then the current increases (Figure 16b). This effect suggests the consideration of the temperature, in addition to the CF gap, as a state variable in line with the approach presented in [100] for second-order memristor. The temperature increase reported above could be employed with a series of pulses to tune the device conductivity in set cycles within a neuromorphic circuit context [100,105,106]. It is noteworthy that a third-order approach has been introduced in modeling devices for neuromorphic engineering [107].

#### 2.4.4. SPICE-Based Circuital Models with Two or More CF Temperatures

In Section 2.3.4, a compact model based on two different CF representative temperatures was reviewed. In that model, some simplifications were performed in order to obtain analytical expressions for calculating these two temperatures although the flexibility linked to the consideration of different temperatures for the CF narrowing and CF main body added high value to the modeling process. In this section, an approach based on the electrical equivalent representation of this thermal framework is proposed in order to calculate numerically the temperature. It is noteworthy that most of the thermal models based on an electrical equivalent circuit (Section 2.4.2 and Section 2.4.3) assume constant thermal resistances and capacitances, which can be used as fitting parameters. However, heat conduction through the CF and lateral heat dissipation depends on the filament size. This effect is included in physically-based simulators, such as those based on a kMC approach or on finite differences/elements, as far as the heat equation is solved using thermal parameters which are updated at simulation time according to the current filament size. Thermal models based on the heat equation analytical solutions in simplified CF geometries (Section 2.3) incorporate also this effect because the temperature analytical expression depends on the actual geometry, and it is evaluated at each simulation time step.

In this section, we present two thermal models based on an equivalent electrical representation (SPICE based), but including the effects of the CF evolution on the thermal properties, which also evolve as the simulation proceeds. The first one is a steady-state model, while the latter includes thermal capacitances. Furthermore, both models account for longitudinal heat conduction and lateral heat dissipation by means of several thermal resistances (or RC networks in the non-steady approach).

**Figure 17:**
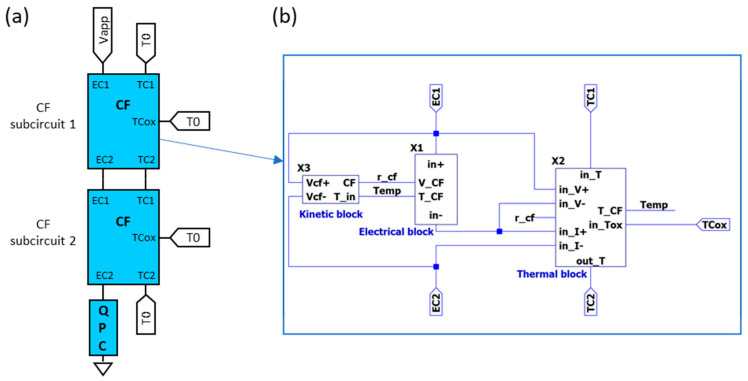
Schema of the circuital compact model. A truncated-cone shaped conductive filament is represented by connected cylinders for modeling purposes (**a**). The behaviour of each portion of the filament (cylinder) is modeled by the subcircuit inside the blue rectangle (**b**), which has electrical connections (EC) and thermal connections (TC). Each cylinder (subcircuit) is characterized by different state variables (radius, r_cf, and temperature, Temp). The cylinder subcircuit consists of several more subcircuits: a kinetic block for calculating the transient CF evolution; an electrical block for current calculation; and, finally, the thermal subcircuit, which includes the equivalent circuit for the thermal model. As can be seen, the subcircuits (thermal, kinetic and electrical blocks) are connected all together because they are interdependent. If necessary, a last subcircuit is added in series (**a**) in order to account for the conduction through a constriction by means of the quantum point contact model (see Section 4) [108].

Figure 17 shows a general schema of the overall model. As explained before, the temperature of the CF main body is, in general, lower than that of the small portion of the filament where the filament evolves faster. Therefore, the CF is modeled by two different subcircuits (Figure 17), each of them characterized by different state variables (CF radius and temperature).

**Figure 18:**
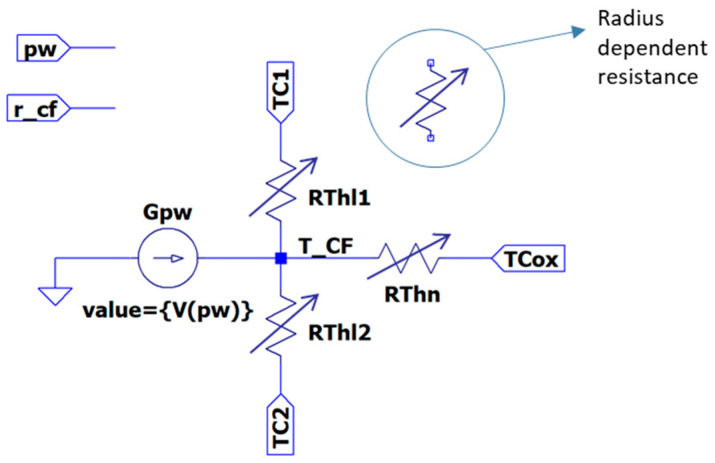
Circuital equivalent for thermal model TM8. The circuit inputs are the dissipated power (pw) and the CF radius (r_cf), while the output is the temperature (T_CF). The actual values of the thermal resistances depend on the filament radius (it is assumed to be a cylinder) and, therefore, they are updated as the CF evolves. The subcircuit has been prepared for being connected to other thermal subcircuits (through TC1, TC2 and TCox) in order to obtain a more complex thermal model of the whole device (with several temperatures along the filament or different temperatures for the surrounding insulator or bulk insulator, Figure 17). If only one block is used, all the resistances are in parallel and the model is equivalent to TM5, although the thermal resistance value keeps the dependency on the filament size in TM8 and it changes during the simulation.

Now, we focus on the description of the thermal subcircuit (Figure 18). The longitudinal heat conduction is modeled by *R_Thl1_* and *R_Thl2_*. For the sake of generality, it has been split off into two contributions in order to make easier the connection with other thermal sub-circuits and to build more complex thermal models. Their values are given by [89]:

(32) $$R_{Thl1i}=R_{Thl2i}=\frac{1}{2}\frac{L_{i}}{k_{th}\;\pi \;r_{i}^{2}}$$

where *k_th_* is the CF thermal conductivity, *L_i_* is the length of the portion of the filament modeled by the sub-circuit and *r_i_*, its radius (index *i* refers to the cylinder or sub-circuit number 1 or 2 in Figure 17a). On the other hand, *R_Thn_* accounts for the lateral heat dissipation and it is calculated following this expression [89]:

(33) $$R_{Thni}=\frac{1}{2\;h\;L_{i}\;\pi \;r_{i}}$$

Note that in this model (TM8), the thermal resistances are not directly the fitting parameters since they are calculated according to the actual filament size. Therefore, as far as the complete device model is able to reproduce the geometrical evolution of the filament (kinetic block in Figure 17), the thermal resistances are not fixed, but their values evolve as the simulation runs. The implementation of such dependent resistances can be made by means of behavioural sources.

Although the model shown in Figure 17 uses two cylinders (and the corresponding two sub-circuits), the CF could be represented by more cylinders in order to get a more detailed CF description [31]. Indeed, in the limit with an infinite number of differential length cylinders, the circuit simulation would be equivalent to the resolution of the differential Equation (9). In fact, with a reduced number of filaments, the results are very similar to those obtained with a finite differences simulator [31,109]. Furthermore, complex filaments such as those with several branches forming a tree structure or interlaced between them could be simulated using more blocks and interconnecting them following a given structure (Figures 5 and 6 in [31]).

On the contrary, if only one block is used, this thermal model (TM8) would be equivalent to TM5, although the thermal resistance evolution is allowed in TM8.

**Figure 19:**
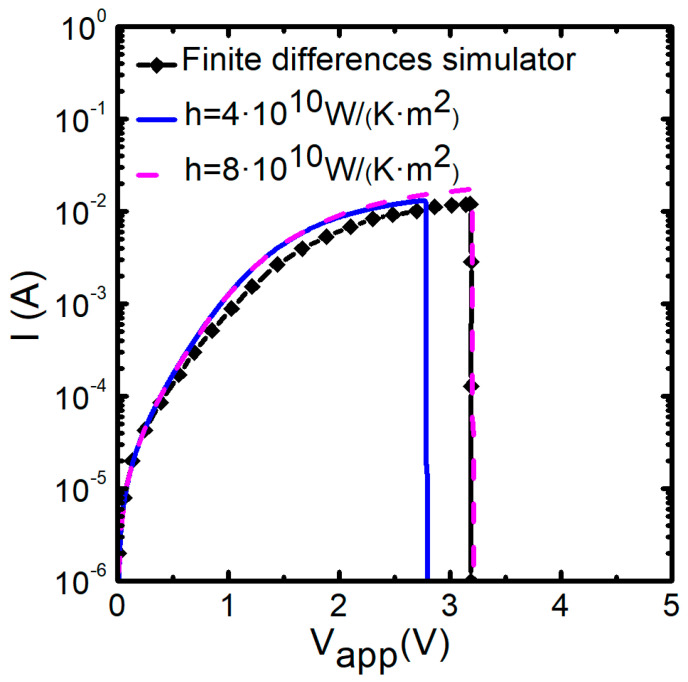
Simulation of a reset transition in unipolar Ni/20 nm-HfO_2_/Si-n^+^ resistive switching devices [109]. The *i-v* curve provided by a finite differences simulator used to fit the experimental data [80] is compared with the *i-v* curve calculated by means of the two cylinders model with TM8. Note that with only two subcircuits (Figure 17a) and taking variable electrical and thermal resistances into account, both types of simulators provide very close results, as far as the circuital model includes variable electric and thermal resistances. Two values of the lateral heat dissipation parameter, h, have been used for the sake of comparison.

Figure 19 shows the results provided by TM8 when is coupled to kinetic and conductive blocks fitted to simulate reset transitions in unipolar Ni/HfO_2_/Si-n^+^ resistive switching devices [80,109,110]. The QPC block has also been added. The two cylinders-TM8 model results have been compared with those provided by a finite differences simulator that was used to fit the experimental data [80]. The lateral heat dissipation parameter, *h*, has been changed in order to check its influence on the *i-v* curve. As can be seen, more heat dissipation requires a higher voltage to reach the thermally triggered reset transition, a well-known effect in RRAMs.

**Figure 20:**
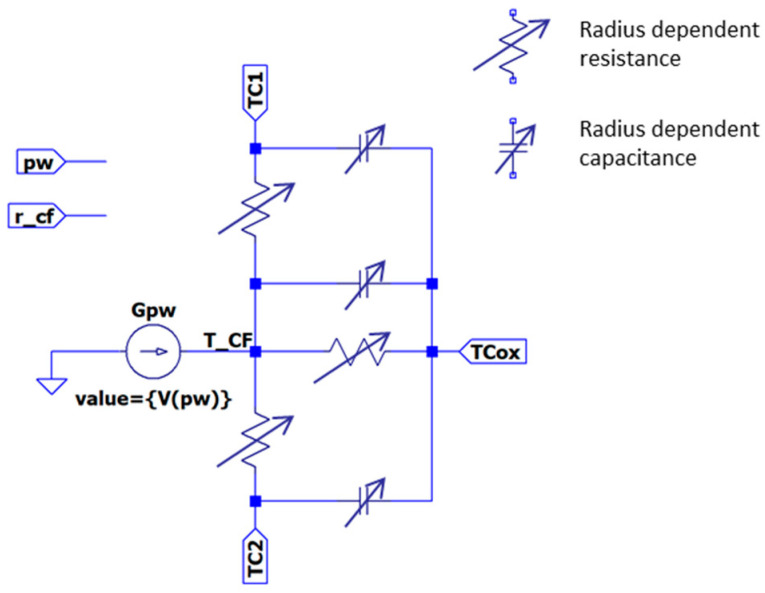
Circuital equivalent for thermal model TM9. It is similar to TM8 (Figure 18), but thermal inertia has been added by means of capacitances. As in TM8, the actual values of the thermal resistances and capacitances depend on the filament radius (it is assumed to be a cylinder) and, therefore, they are updated as the CF evolves [37].

Thermal inertia can also be considered following this approach if thermal capacitances are added (Figure 20, thermal model TM9) [37]. In this new model, thermal capacitances could also be made dependent on the filament size instead of being fixed fitting parameters [37,111]. As previously mentioned, several blocks can be connected for modeling the CF. On the contrary, if only one segment is used, the model is equivalent to model TM6 (although TM9 lets the thermal components evolve at simulation time).

**Figure 21:**
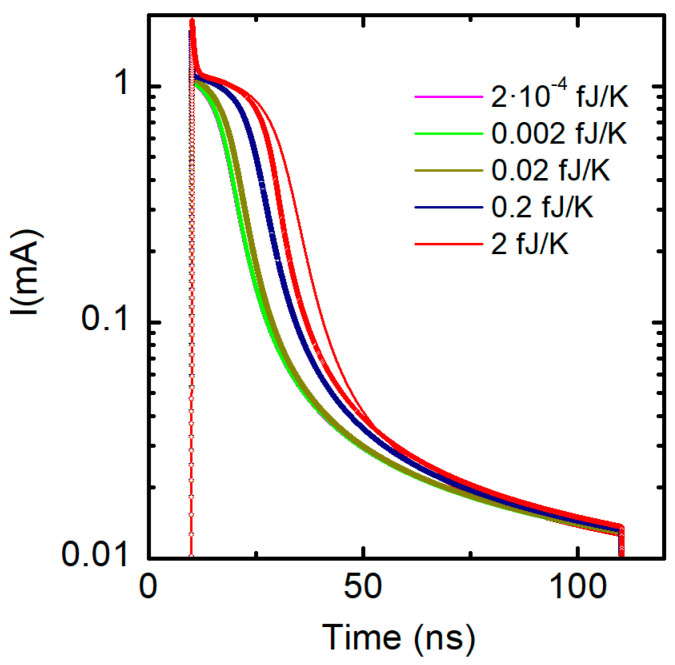
Simulated current in a Ni/20 nm-HfO_2_/Si-n^+^ resistive switching device [37,110] when a 3 V reset pulse is applied (for 100 ns). Different values of the thermal capacities have been assumed. Fixed thermal capacitances (solid lines) and size-dependent thermal capacitances (symbols) have been used [37,111].

Figure 21 shows the simulation of the transient response of a Ni/20 nm-HfO_2_/Si-n^+^ resistive switching device [110] when a 3 V reset pulse is applied (for 100 ns) [37]. Several values of the thermal capacities have been considered. The simulation context here is different from the one shown in Section 2.4.3 since all the modeling components are linked to the device conductive filaments. Note also that only values higher than 0.2 fJ/K influence the device response. Fixed and variable thermal capacities have been used in order to analyze the role of size-dependent thermal capacities, which evolve at simulation time. As expected, variable thermal capacitors, whose value is reduced during a reset process, produce lower thermal inertia.

Finally, it was previously seen that TM7 deals with two temperatures (at the filament and at the surrounding insulator). Note that although TM9 (following the two cylinders schema shown in Figure 17) also consider two temperatures, they are both linked to the conductive filament. TM7 can be obtained as a particular case of TM9 considering only one cylinder, but connecting another RC network between the node *TCox* and a voltage source for representing the bulk insulator temperature. It is important to note that in the TM9 case, variable (at simulation time) thermal components are allowed.

## 3. General Memristor Modeling Framework with Thermal Effects Emphasis

In this section, a different approach to model RRAM thermal features is proposed. For this purpose, we make use of the general memristor modeling workspace that was introduced by Corinto and Chua in [71]. This alternative perspective complements the developments presented in the previous section. In particular, the authors [71] developed a unified theoretical framework and also discussed the advantages of using the flux-charge (φ-Q) domain to study memristor elements. Within this framework, memristors, in the taxonomy proposed in [72] (the ideal, the generic, and the extended memristor), are described as the result of different approximations in the equations. This extended categorization emerged as a necessity in order to include theoretically the description of pinched, hysteretic behaviours demonstrated by various elements, not only in circuit theory and electronics but also in nature.

Among the different categories presented above, the most general class is linked to extended memristors, which refers to memristors that have extra state variables (in addition to φ and Q). For the specific case of charge-controlled memristors, they are described by the following equations:

(34) v=M(Q,i,X) i

(35) $$\frac{dX}{dt}=g_{Q}\left(Q,i,X\right)$$

(36) $$\frac{dQ}{dt}=i$$

The memristance *M* of an extended memristor is implied in Equation (34), apparently bearing the feature of nonlinearity; *v* is the voltage across the memristor, *i* is the current flowing through it, and *Q* is the charge, i.e., the current first momentum. The vector ***X*** stands for a set of extra state variables, including all the necessary physical magnitudes according to the implemented memristive system; indicatively, they could be the device internal temperature, the conducting filament radius, or any other non-electrical variable influencing the memristor state that ultimately affects the device charge conduction. Apparently, the dynamics of the state variables ***X*** are governed by *g_Q_*(*Q*,*i*,***X***) and Equation (35). It is noted that the importance of the class of extended memristors comes from the fact that all real-world memristor devices known until now are indeed extended memristors. Notice that from Equation (34), we can define the memristance as follows:

(37) $$M\left(Q,i,X\right)=\frac{v}{i}=\frac{d\varphi /dt}{dQ/dt}=\frac{d\varphi }{dQ}$$

where φ is the voltage first momentum, usually called flux in analogy with the charge. See, however, that a more useful way to obtain this relation is through derivation by assuming that the charge and the flux are related by a function *f*:

(38) φ=f(Q,i,X)

Then, by deriving with respect to time, and using the chain rule, we obtain:

(39) $$\frac{d\varphi }{dt}=v=\frac{\partial f\left(Q,i,X\right)}{\partial Q}\frac{dQ}{dt}+\frac{\partial f\left(Q,i,X\right)}{\partial i}\frac{di}{dt}+\frac{\partial f\left(Q,i,X\right)}{\partial X}\frac{dX}{dt}=\frac{\partial f\left(Q,i,X\right)}{\partial Q}i=\frac{\partial \varphi }{\partial Q}i$$

The latter part of Equation (39) is obtained under the assumption [71] described below,

(40) $$\frac{\partial f\left(Q,i,X\right)}{\partial i}\frac{di}{dt}+\frac{\partial f\left(Q,i,X\right)}{\partial X}\frac{dX}{dt}=0$$

The system memory capability under no excitation is determined by a special case of Equation (35), often referred to as the power-off plot (POP) equation; in this case we have *i* = 0 or equivalently *Q* = constant. If a single state variable is considered, it is clear that if the POP equation is nil under these conditions, the system presents then a long-term memory since the state variable will not change with time; while if it is different to zero, the system is capable of exhibiting only short-term memory.

The consideration of the temperature, *T*, as the state variable is a peculiar case since there is an influence from external sources (i.e., the ambient –room– temperature, *T_0_*); this implying a possible energy input in some cases if *T_0_* is not constant. In addition, as it has been discussed in Section 2, it is difficult to determine a single value for the device internal temperature (some models, as shown above, include two different temperatures to better describe the device operation). We can write the equations by separating the temperature as follows:

(41) $$v=M\left(Q,i,X,T,T_{0}\right)\;i$$

(42) $$\frac{dX}{dt}=g_{Q}\left(Q,i,X,T,T_{0}\right)$$

(43) $$\frac{dT}{dt}=g_{T}\left(Q,i,X,T,T_{0}\right)$$

These equations include both temperatures: the internal device temperature, *T*, and the external *T_0_*. Obviously, there is no equation governing *T_0_* dynamics since it can be considered as an external signal. Temperature, *T*, may be a position-dependent temperature T(x,y,z), as already presented in Section 2.1. In this case, Equation (43) would correspond to the heat equation. As an additional note, it is important to highlight that a device will not present long-term memory characteristics associated solely with temperature, since the device will tend to reach thermal equilibrium with the external medium. In absence of any external electrical input, this would mean that the POP equation related to the evolution of the internal temperature is not zero in the general case. This does not preclude, however, that the system may have other internal variables that do present a long-term memory capability. As an example, we can think if the case of a phase change memory (PCM), where a phase change is activated by temperature, and it remains even after the device has cooled back to room temperature. A similar situation occurs when a RRAM conductive filament is ruptured because of an enhanced diffusion process favored by a temperature rise [24,80].

### Example of Application

As an easy example, we can consider the case of the thermistor, which is one of the first elements identified as an extended memristor, i.e., a device whose resistance is dependent on some internal state variable that presents memory effects [112,113]. In this respect, and for the modeling developments henceforth, they display parallelism with RRAMs.

The model has been known since Steinhart and Hart published a function which fitted the variation of thermistor-resistance according to temperature [114] as a Taylor’s expansion of the device conductance in terms of the temperature logarithm. This function, along with the equivalent Ohm’s law in Equation (44), has proved to be suitable in a wide variety of thermistors, for ranges from a few degrees to a few hundred degrees, and it has been widely used to model this kind of devices when used as temperature sensors. The most usual way to describe it is by means of a simplification shown in Equation (45). In addition, a key thermistor characteristic is linked to self-heating, which can be described by Equation (46), by neglecting radiative heat dissipation:

(44) $$v=M\left(T,T_{0}\right)\;i$$

(45) $$M\left(T,T_{0}\right)=R_{0}\;exp\left(B\;\left(\frac{1}{T}-\frac{1}{T_{0}}\right)\right)$$

(46) $$\frac{dT}{dt}=\frac{R_{0}}{C}i^{2}-\frac{\delta }{C}\left(T-T_{0}\right)$$

In the above equations, *T*_0_ is the room temperature, and *T* the device internal temperature. The rest of the symbols are parameters of the thermistor model and can be considered as constants for all practical purposes. At this point, it is noteworthy to point out that these equations bear exactly the same form as Equations (41) and (43) and, thus, identify the thermistor as a memristor. That is, the thermistor is a device whose resistance depends on its electrical history, and it has an internal state variable that governs the overall behaviour (the device internal temperature). Thus, the device can be classified as an extended memristor.

In addition, if we look at the POP equation (Equation (46) with *i* = 0), we see that the temperature derivative is different from zero for any situation other than the device thermal equilibrium with the surrounding environment. Thus, the device does not possess a feature linked to long-term memory. In this respect, it is also illustrative to point out that there are other memristive systems [115] that are also extended memristors due to self-heating, even if the thermal specific mechanisms are different from those of thermistors.

**Figure 22:**
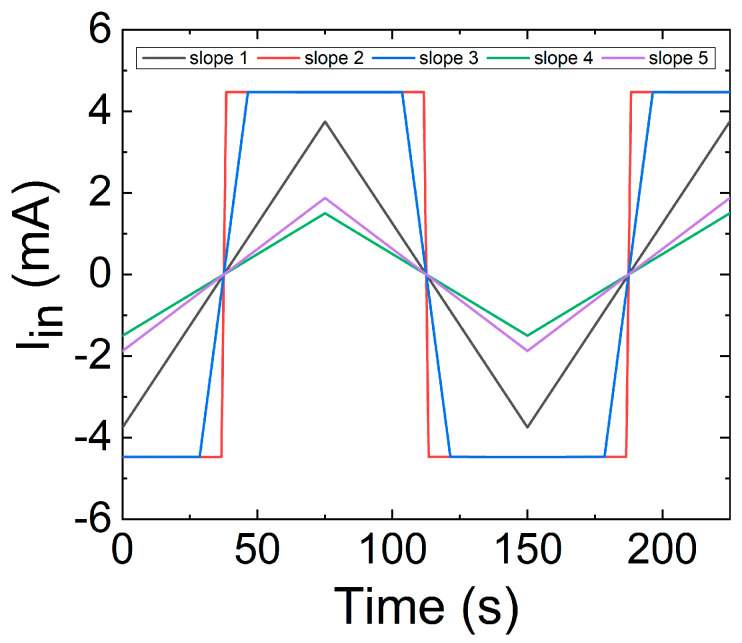
Input current waveform versus time for five different ramps. Colours are coherently employed in the following plots.

As an example to illustrate the memristive behavior of this device, we have simulated it when driven by a triangular current waveform, using specific values extracted from a datasheet for the thermistor constants: *δ* = 4 × 10^−3^ W K^−1^, *C* = 60 × 10^−3^ J K^−1^, *B* = 3950 K, *T_0_* = 298 K, *R_0_* = 10 kΩ. Additionally, and in accordance with a typical thermistor datasheet, we have set a maximum current of 4.5 mA, and we have also used 5 different ramp slopes, as plotted in Figure 22.

**Figure 23:**
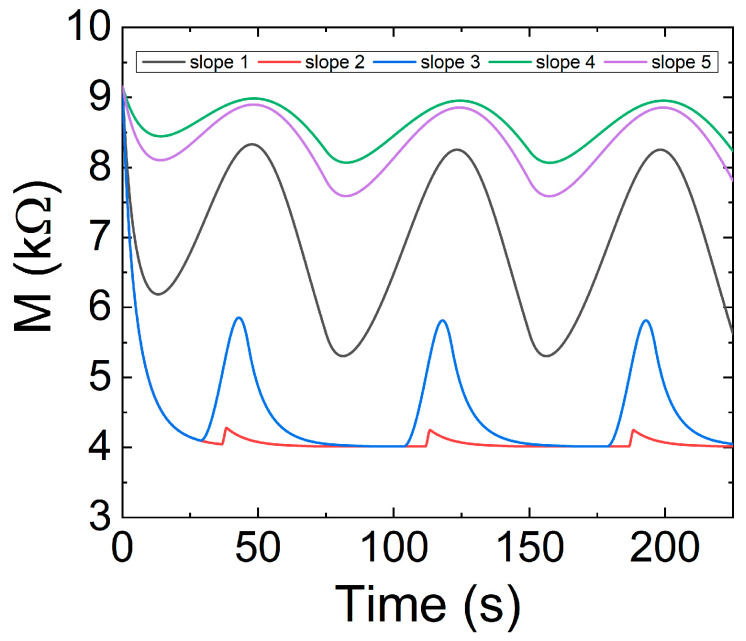
Memristance versus time for five different input voltage signals under ramped voltage stress.

Figure 23 plots the evolution of the memresistance as well as the current input versus time. The input signal shown in Figure 22 has been employed as well as the Equations (44)–(46).

**Figure 24:**
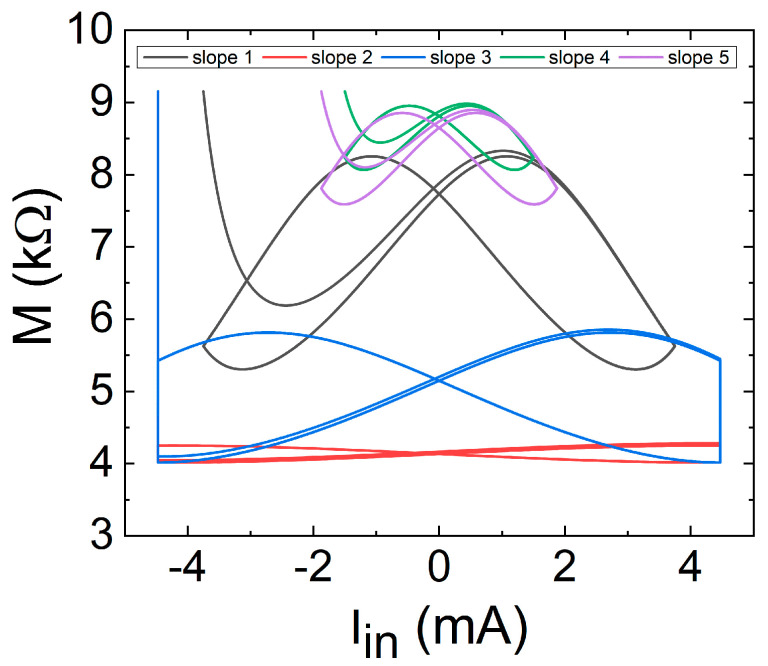
Memristance versus input current, for five different slopes. Colours are coherent with the results shown in the previous figures.

In addition, we have also plotted these two magnitudes used in the *Y* axis previously against each other in Figure 24, showing the effects of the different slopes in the device memristance.

**Figure 25:**
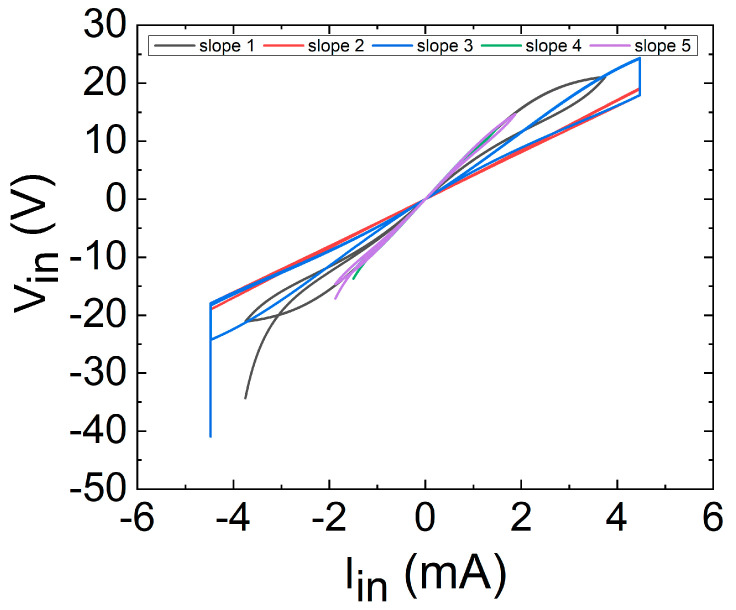
*i-v* thermistor characteristics for five different input voltages.

Then, the typical *i-v* memristor characteristics showing the famous loop are drawn in Figure 25. See that the shapes are in line with what is expected for memristors [71].

Figure 25 shows the two fingerprints of a memristor [116]: (1) a pinched loop; and (2) an area that varies with frequency, tending to a line at high frequencies. In fact, the behaviour at the highest frequency (cyan line) is nearly that of an ohmic resistor, with no loop area, while at lower frequencies the behaviour is different. It has to be noted that (see [115]) the situation is more interesting than simply an area increase at lower frequencies. As highlighted in [115], the thermistor control variable is the internal temperature and it tends to be in thermal equilibrium with the surroundings, which causes the loop area to reduce at a very low frequency. As we use it in the corresponding equations, we can see that for very low input signal slopes, the device always reaches thermal equilibrium, since the *dT*/*dt* is nearly zero, and its behaviour tends to be close to a nonlinear resistor, as shown in Figure 24; Figure 25 (red lines), with a null area enclosed in the loop.

**Figure 26:**
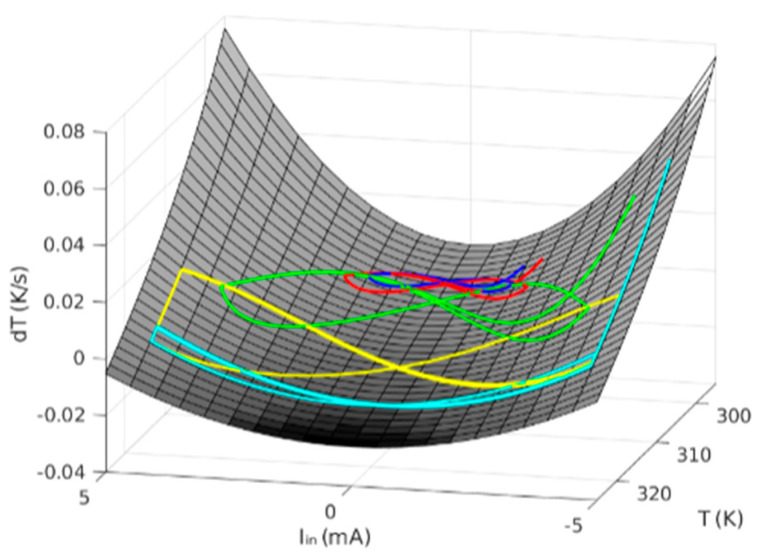
Memristor Dynamic Route Map (surface), showing as lines the five trajectories corresponding to the previous figures, using the same colour code. It can be seen that all these trajectories fall on the surface, which defines univocally the device behaviour. Notice that this surface is, in fact, a family of surfaces that depend on the room temperature *T_0_*.

At this point, the concept of dynamic route map (DRM) [36] comes up since it is quite useful to represent the device time evolution (again, in this facet, a full parallelism with RRAM is observed [36]). In fact, the DRM is a concept arising from non-linear dynamical systems, and represents the trajectories a system follows in the phase space of the state variable versus its derivative for different control parameter values. If we plot it as a 3D diagram, we find that all the trajectories, for a given constant *T_0_*, are bound to fall on the same surface, as seen in Figure 26. Using this representation may provide very interesting insights into the device dynamics. Considering our thermistor, if a trajectory goes from a state with positive derivative to another characterized by a negative derivative with increasing temperature, then it will reach a stable equilibrium point at the temperature where the derivative nullifies. An equilibrium point is a state where the device tends to remain at even if it drifts from it in its operation; this idea resembles a similar concept related to the DC quiescent point in circuit theory, or memory in memristors. In the opposite case, when the trajectory goes from negative to positive, an equilibrium point might seem to come up at the zero-crossing temperature, but it is unstable, which means that the slightest change will force the system to come out of it.

## 4. RRAM Quantum Point Contact Modeling, Thermal Effects

So far, we have studied thermal effects from a classical physics viewpoint. This is the approach commonly used in most compact models. However, the scale of the material layers that form part of a RRAM makes feasible for certain devices and for particular operation conditions the observation of quantum effects. In particular, when the conducting filament (CF) cross-sectional area in a RRAM device becomes very narrow, only a few atoms wide, the continuum description is expected to break down, so that the direct application of the heat equation becomes questionable [117,118]. We enter into the regime of heat transport and dissipation at the nanoscale [119]. Before starting with a discussion about these topics, it is important to discriminate between the role that temperature plays on the ion/vacancy movement across the insulating films (essentially governed by Kramers’ theory [120]), and the temperature dependence of the mechanism adopted for the electronic transport in the CF itself (Schottky, Poole-Frenkel, tunneling, variable range hopping, space charge limited conduction, quantum point contact, etc.) [121]. Although both descriptions are intrinsically connected and they are at the heart of the complexity of the RRAM behavior, they are often treated separately for simplicity, or one of them completely dropped. To deepen into these intertwined approaches, a detailed RRAM dynamic simulation at the microscopic level is imperative. Ion/vacancy diffusion process, which defines the filamentary structure, depends on temperature, and the size of the structure determines the magnitude of the electron current that governs the power dissipation (Joule heating effects), which in turn affects the local temperature that drives the diffusion process. In this section we will exclusively focus the attention on the modeling of the electron transport at the nanoscale and the influence of temperature and power dissipation on it. The role of ions/vacancies will be indirectly addressed (more on this issue is explained in Section 5). Temperature evolution in the structures under study (e.g., Figure 1; Figure 2) is extensively covered in Section 2. First, a fixed ion/vacancy arrangement will be considered for simplicity, so that two particular extreme cases, LRS and HRS, will be examined. Second, a phenomenological model for the transition from HRS to LRS which takes into account the power dissipated at the CF bottleneck will be presented. As already discussed previously, while in LRS, the CF is completely formed establishing a metallic-like connection in between the electrodes; in HRS, the filament presents a gap along its structure as a consequence of the absence of conduction states [122]. Thereby, a common theory for both cases able to demonstrate consistency with the experimental observations is required.

Mesoscopic physics, a subdiscipline of condensed-matter physics, has resulted in a suitable framework for semi-empirically describing the temperature dependence of the electron flow in narrow constrictions and, in particular, in RRAMs. Mesoscopic physics describes the conducting properties of systems whose size lays in between the macroscopic (bulk material) and the microscopic (atoms and molecules) worlds. Since we are talking about atomic dimensions, quantum mechanics is at the foundations of mesoscopic physics [123]. Within this framework, the quantum confinement associated with filamentary conduction is described in terms of the electrochemical potential, potential wells, potential barriers, and bands (valence, conduction, gap). We also talk about a semi-empirical approach because the local temperature is frequently unknown and the external temperature is considered as a control parameter. This objection can be partly overcome if models including two different temperatures (for the cold and hot part of the CF, and for the CF and its surrounding region, see Section 2.3.4 and Section 2.4.3) are employed. The idea of using a mesoscopic approach is the consequence of the observation of experimental RRAM conductance values around integer and non-integer multiples of the quantum conductance unit *G*_0_ = 2 *e*^2^/*h*, where *e* is the electron charge and *h* the Planck’s constant [124]. In terms of resistance, this unit is *R*_0_ = 1/*G*_0_ = 12.9 KΩ. The experimental conductance values for many cycles measured at a fixed bias, or the conductance measured at consecutive steps in one cycle at different or constant biases are often displayed using histogram plots with the x-axes normalized to *G*_0_. In many cases, these histograms reveal a peak structure which is interpreted as an indicator of the number of channels available for conduction or as the occurrence of preferred atomic configurations for the CF [125]. Although the detection of peaks in the device histograms is recognized as the signature of quantum point-contact conduction, it should be taken into account that measurements can be seriously affected by a number of factors such as the existence of multiple conduction paths, series resistance, roughness and scattering caused by the granularity of matter, in general, non-adiabatic (non-smooth) potential profiles. Caution should also be exercised with the use of the term *conductance quantization*: only for simple *s*-electron metals, the transmission probability for the conductance channels is expected to open close to integer values [126]. For this reason, observations in the field of RRAM should be more appropriately considered to be in the quantum (rather than in the quantized) regime of conductance [125].

Many experimental results on resistive switching materials have been interpreted in terms of conduction through atom-sized filamentary structures [127]. This is the case of a wide variety of binary and ternary oxides such as SiO_x_ [128,129,130,131], HfO_2_ [132,133,134,135,136,137,138,139], Ta_2_O_5_ [140,141,142], NiO [143,144], ZnO [145,146], a-Si:H [147], TiO_2_ [148], V_2_O_5_ [149], YO_x_ [150], and BiVO_4_ [151]. Nonlinear effects in HfO_2_ were also reported by Degraeve et al. [152] and in CeO_x_/SiO_2_-based structures by Miranda et al. [153]. From the point of view of theory, it is worth mentioning that the CF formation in monoclinic- and amorphous-HfO_2_ was investigated from first principles by Cartoixa et al. [154] and by Zhong et al. [155]. The filamentary paths are built from oxygen vacancies and using a Green’s function formalism coupled to a density functional theory code, the conductance of filaments of different lengths was calculated. According to the obtained results, even the thinnest CFs can sustain conductive channels exhibiting signs of quantum conduction.

Very often, LRS is associated with conductance values *G ≥ G*_0_ and with a linear *I-V* curve (not to be confused with Ohmic behavior). In this case, the device conductance can reach values from 10 to 100 times *G*_0_ which indicates the large number of atoms participating in the filament formation. On the other hand, HRS is associated with conductance values *G* < *G*_0_ and with a non-linear *I-V* curve (mainly with exponential behavior). This state is characterized by a gap or potential barrier which acts as a blocking element for the electron flow. As the starting point for the inclusion of the thermal effects in RRAMs, the Buttiker-Landauer approach for quantum point contacts is considered [156]. Importantly, the analysis does not discriminate between CBRAMs and OxRAMs, so they are treated on equal grounds.

**Figure 27:**
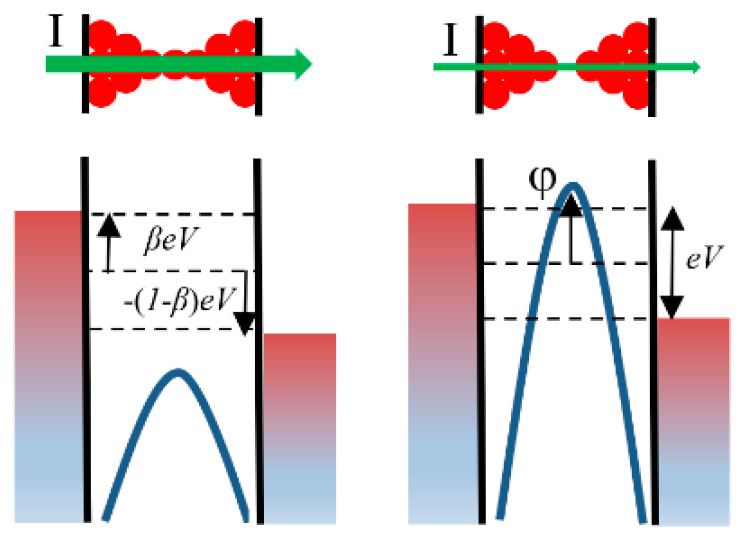
Schematic of the energy structure of the conducting filament. In LRS (high current), the CF is completely formed and the confinement potential barrier is low. In HRS (low current), the filament is broken and the confinement potential barrier is high. The green arrows width indicates the electron current magnitude.

According to the finite-bias Landauer’s formula [157], the *I-V* characteristic of a mesoscopic conductor can be expressed as:

(47) $$I=\frac{2e}{h}\int^{\;}D\left(E\right)\left[f\left(E-\beta eV\right)-f\left(E+\left(1-\beta \right)eV\right)\right]dE$$

where *E* is the energy, *D* the tunneling probability, *f* the Fermi-Dirac (FD) distribution function, and 0 ≤ *β* ≤ 1 the fraction of the applied voltage that drops at the source side of the constriction. For a symmetrical structure *β* = ½. Assuming an inverse parabolic potential barrier for the constriction bottleneck, *D* is given by [158]:

(48) $$D\left(E\right)={\left\{1+exp\left[-\nu \left(E-\varphi \right)\right]\right\}}^{-1}$$

where ***ν*** is a coefficient related to the curvature of the potential barrier and *φ* the height of the potential barrier that represents the confinement effect (see Figure 27). For *T* = 0 K, (47) and (48) yield [159] (see Figure 27):

(49) $$I\left(V\right)=G_{0}\left\{V+\frac{1}{e\nu }ln\left[\frac{1+exp\left[\nu \left(\varphi -\beta eV\right)\right]}{1+exp\left[\nu \left(\varphi +\left(1-\beta \right)eV\right)\right]}\right]\right\}$$

**Figure 28:**
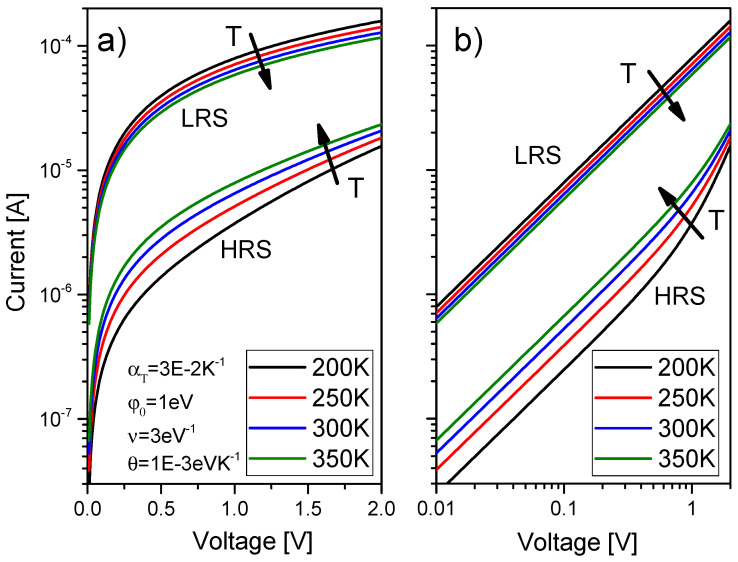
Effects of the temperature on the HRS and LRS I-V characteristics. (**a**) log-linear axis and (**b**) log-log axis.

Figure 28 shows some typical modeling results using Equation (49). Any additional potential drop along the confinement structure can be accounted for using the transformation *V→V-I R*_S_ in (49), where *R*_S_ is a series resistance. Equation (49) can be modified so as to include many parallel conducting channels [138]. For LRS, we can consider that there is no blocking element along the CF so that assuming *φ*→−∞ (*D*→1) in (49), we obtain:

(50) $$I\left(V\right)=G_{0}V$$

which is the celebrated Landauer formula for a monomode ballistic conductor [160]. Following [134], the temperature dependence can be introduced into (50), assuming *R*_S_(*T*) = *R*_S0_·[1 + *α_T_*(*T − T*_0_)], where *R*_S0_ = *R*_S_(*T*_0_), *α_T_* is a temperature coefficient, and *T*_0_ the room temperature. In this case, the *I-V* characteristic still follows a linear relationship but with a lower slope given by:

(51) $$I\left(V\right)=\frac{G_{0}}{1+G_{0}R_{S}\left(T\right)}V$$

If *α_T_* is a positive coefficient, as expected for a metallic-like conductor, the current decreases as the temperature increases (see Figure 28). This behavior is in agreement with the experimental observations [134]. Notice that here the emphasis is put on the connection of the ballistic region with the rest of the device (internal or external) and in particular with the contacts. Nevertheless, *R*_S_ can also be viewed as the momentum relaxation factor along the filamentary structure. If we move to the opposite limit, for HRS, and we consider specifically the case *E* << *φ*, (48) reads:

(52) D(E)≈ exp[ν(E−φ)]

so that (47) can be integrated taking into account the temperature-dependent smearing of the FD distributions at the contacts. The result is [161]:

(53) $$I\left(V\right)\approx \;\frac{2e}{h\nu }\;\frac{exp\left(-\nu \varphi \right)}{sinc\left(\pi \nu kT\right)}\left\{exp\left[\nu \beta eV\right]-exp\left[-\nu \left(1-\beta \right)eV\right]\right\}$$

which provides the exponential behavior observed for HRS. Now, notice that the temperature appears explicitly in (53) through the *sinc* function. In this case, the current increases with the temperature as expected from the availability of more energetic electrons at the injecting electrode. However, it can be shown that for a set of typical fitting parameters, the smearing of the FD functions is not enough to account for the observed temperature effects in HRS. In order to circumvent this problem, a new parameter is introduced into the model Equation (53) so that a larger variation of the current can be achieved. Following experimental observations for the soft breakdown conduction mode in SiO_2_ [161], the confinement potential barrier height *φ* can be parameterized as *φ(T) = φ_0_-θ(T − T_0_)*, where *φ_0_*_=_ *φ(T_0_)* and *θ* > 0 is a linear temperature coefficient. This correction term arises from the thermal movement of ions/vacancies in the CF around their equilibrium positions. In this case, as the temperature increases, the tunneling current increases because of the reduction of the effective barrier height (see Figure 28). The temperature effect on the barrier profile was recently investigated in detail in [139] using inverse modeling in combination with the WKB approximation for the tunneling probability.

To conclude this section, it is worth mentioning that according to the standard theory of mesoscopic devices, heat largely dissipates at the electrodes (reservoirs) and thermal and electrical conductances are proportional through the Wiedemann-Franz law [119]. This idea is to heuristically explain why a CF of atomic dimensions is able to reach a stationary current state with conductance values of the order of *G*_0_. Notice that the current density flowing through a nanoscale CF can be extraordinarily high. The question can be summarized as, where is power dissipated in a RRAM system exhibiting quantum properties? This is a fundamental question in mesoscopic physics [123]. Let us consider here the progressive increase of the current flow as a function of time when the device is subjected to a constant voltage stress after electroforming. This process corresponds to the transition HRS→LRS which arises because of the CF widening. Following [162], we can write first the following phenomenological equation for the current evolution:

(54) $$\frac{dI}{dt}=\eta P_{C}$$

where *η* is a temperature- and material-dependent coupling coefficient and *P_C_* is the power dissipated at the constriction bottleneck. For the simplest case of a constant applied bias *V*, Equation (54) expresses that the current levels off in the long run because power dissipation first increases and then progressively transfers from the constriction to the electrodes. Second, according to Landauer’s formula, the transmission probability *D* (average) can be expressed as a function of the current flowing through the structure as:

(55) $$\tilde{D}=G_{0}^{-1}G={\left(G_{0}V\right)}^{-1}I$$

and the power dissipated at the constriction can be calculated from the voltage drop *V_C_* occurring at the constriction using:

(56) $$P_{C}=V_{C}I=V\left(1-\tilde{D}\right)I=VI\left(1-\frac{I}{G_{0}V}\right)$$

Then, from expression (54), an explicit differential equation for the current evolution is obtained:

(57) $$\frac{dI}{dt}=\eta VI\left(1-\frac{I}{G_{0}V}\right)$$

**Figure 29:**
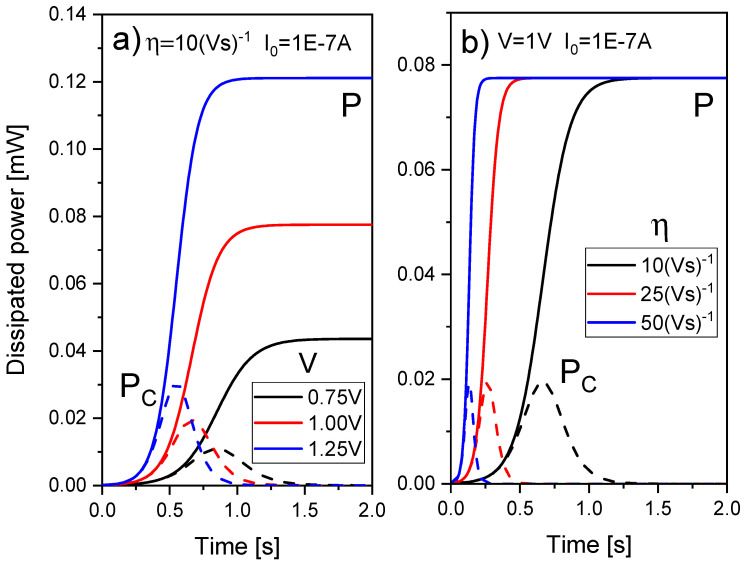
Evolution of the power dissipated in the structure P (solid lines), and at the constriction P_C_ (dashed lines). (**a**) Corresponds to different applied voltages *V*, and (**b**) corresponds to different transition rates *η*.

Equation (57) is nothing but the logistic equation with effective transition rate *ηV* and carrying capacity *G*_0_*V*. The solution to Equation (57) for a constant bias reads:

(58) $$I\left(t\right)=\frac{G_{0}I_{0}Vexp\left(\eta Vt\right)}{I_{0}\left[exp\left(\eta Vt\right)-1\right]+G_{0}V}$$

which complies with *I*(*t = 0*) = *I_0_* and *I*(*t* = ∞) = *G*_0_*V*, the initial and stationary conditions, respectively. Equation (58) expresses that, when a mesoscopic channel with conductance *G*_0_ is formed, the power fundamentally dissipates at the electrodes and not at the constriction’s bottleneck (see Figure 29). Power is indeed dissipated at the constriction during the CF formation as discussed in the next section. Of course, this is a simplistic view of a much more complex process.

Although most of the models addressed in this manuscript are devoted to devices that show single filamentary conduction, some of them could also be applied to area-dependent devices and even to devices that do not require electroforming [1,2,70]. In particular, the Landauer’s approach can be extended to area-dependent devices by assuming multifilamentary conduction. This is the case when the Landauer formula (49) includes a prefactor N dealing with the number of identical filaments assumed [138,159]. In addition, a modeling procedure in line with the general memristor framework presented in Section 3 could also be possible [71,72].

## 5. Thermometry of Conducting Filaments

In addition to the developments described above in relation to the HE solutions in different modeling approaches and levels of complexity, it is interesting to understand the dielectric breakdown (BD) phenomenon in the context of RRAM operation. In this respect, we shed light in this section on the structural damage that occurs during the BD current transient. In ultra-thin dielectric layers (<5 nm), there is a wide consensus around considering that the intrinsic BD is related to the generation of defects in the dielectric film [163,164,165,166]. When the density of bulk defects is high enough, the BD event is triggered by the local connection of the electrodes through a defect related conduction path. Once a defect percolation path is formed, the main feature is a progressive increase of the current across the dielectric. This phenomenon, often referred to as progressive breakdown (PBD), is an universal process that lies under a wide variety of dielectric materials, ranging from traditional oxides, such as SiO_2_, SiO_X_N_Y_ [167] to innovative 2D dielectrics, such as h-BN [168], passing through high-k materials such as Al_2_O_3_ and HfO_2_ [169].

The physical structure of the filament formed during the PBD regime has been deeply studied. In the case of poly-Si/SiON/Si MOS devices during PBD, it was demonstrated that the filament is, at least in part, made of Si atoms, through the mechanism of dielectric breakdown induced epitaxy (DBIE) [170,171]. The filament sizes were directly observed by scanning transmission electron microscopy (STEM) after the BD of MIM structures with either Ti/HfO_2_/TiN or with Hf/HfO_2_/TiN devices, in which the top electrode (Ti or Hf) acted as cathode. Clear evidence of the formation of a metallic filament made of, respectively, Ti or Hf was reported by using electron energy loss spectroscopy (EELS) imaging [172,173]. In the case of 2D h-BN (CVD) dielectric layers, there is strong evidence that the CFs are formed by metal ions that penetrate from the electrodes into the h-BN stack under the action of the electric field [174,175].

Different experiments have probed that the SET event in RRAM devices [176,177] and the dielectric BD of gate oxides [169,178] have some common aspects. In addition to the clear dependence of the CF characteristics on the maximum current flowing through the device [18,179], TEM imaging of Si-based MOS capacitors prior to and post dielectric BD [163,170,180] and HfO_2_-based RRAM cells after forming and cycling [172,181] show comparable microstructural changes in the oxide, suggesting the diffusion of the anodic atomic species into the oxide layer in both cases. Thus, these two phenomena share not only similar electrical characteristics, but also generate comparable microstructural changes, suggesting a common underlying physical mechanism. In such scenario, we propose to model the results for the SET event in RRAM devices similarly to the gate-oxide BD in MOSFETs.

The PBD effect has been captured by the model proposed by Palumbo et al. in [169,182], and later expanded by Lombardo in [183], clarifying the primary role played by the carrier energy loss through the PBD spot. Such model accounts for the physics behind the progressive evolution of the current, where the BD process is closely linked to the energy transfer from the CF itself to its surrounding atomic lattice. According to this idea, the high temperature associated with the localized current flow (being the BD spot area of 1–50 nm^2^, the current density can reach a few MA/cm^2^ [184,185,186]) would contribute to the generation and enlargement of the BD filament connecting the electrodes of the stack, enabling the promotion of the electro-migration of the fastest available atomic species. Since this technique unambiguously relate the transition rate (*dI_Tr_/dt*) to the heat dissipation properties during the atomic diffusion of the cathode or anode atoms into the gate dielectric in the region of the percolation path, it is possible estimate the CF temperature. Considering the model reported in [169], we can express the current transition rate (marked as *TR*) as:

(59) $$TR=\frac{dI_{Tr}}{dt}=\frac{q\;V\;f_{1}}{k_{B}\;T\;t_{ox}^{2}}DI_{SET}$$

where *T* is the temperature of the CF, *t_ox_* is the dielectric thickness, *k_B_* is the Boltzmann constant, *D* is the diffusion constant of the atomic species responsible for the generation of CF, *I_SET_* is the current level at the onset of the transition, and *f*_1_ = *n_e_λ_e_σ_e_*, with *n_e_* being the electron density, *λ_e_* the electron mean free path and *σ_e_* the cross-section for the electron-atom collision (responsible for the momentum transfer). *V* is the applied voltage across the BD spot which has been assumed to be equal to the overall externally applied bias between the metal contacts of the stack. *f*_1_ value is around the unity since the defect concentration in the CF is most likely very high [172]. According to Equation (59), *dI_Tr_/dt* is proportional to *D* × *I_SET_*. This means that the BD growth rate rises either by increasing the dominant diffusivity D of the fastest atomic species or by increasing the charge carrier flux.

The *I-V* characteristics under the PBD regime can be explained by some well-known physical models, for example invoking trap-assisted tunneling (TAT) current [187], co-tunneling [188,189], and the quantum point contact model [159]. Although the transport properties of stacks with different materials are fitted by considering different transport models, the underlying concept is similar in all cases, the electrons passing through the PBD spots experience a very large energy loss. To simplify the approach, the BD spot *I_SET_*-*V* curve is usually modeled by assuming a simple analytical dependence as described in [190].

It is important to mention that independent experiments have probed that power dissipation taking place inside the dielectric layer is a reasonable assumption. According to Takagi [191], electrons tunneling through defects responsible for stress induced leakage current (SILC) in thin oxynitrides loose a fraction of energy, and as shown by Blochl and Stathis [192], this is caused by defect relaxation. It is reasonable to assume that a similar effect takes place for electron transport through the BD spot, since there is a clear dependence of the CF characteristic on the maximum current flowing through the device, both for the BD of gate oxides [179] and the SET event in RRAM devices [18,163,193], as well as for layered dielectrics, such as h-BN [168,190].

A simplification of spherical symmetry around the CF can be assumed to model the temperature in the BD spot. Within this approach, the temperature can be described by Equation (60), where the dissipated power at the CF is proportional to *I_SET_* × *V*, *k_th_* is the thermal conductivity of the dielectric, *T*_0_ is the room temperature and *f*_2_ is the fraction of the energy lost at the constriction:

(60) $$T=\frac{f_{2}\;V\;I_{BD}}{2\;\pi \;t_{ox}\;k_{th}}+T_{0}$$

The fitting parameters are *f*_1_, *f*_2_, *D_0_*, and *E_act_*; (taking into consideration that D = D_0_*exp(-E_act_/k_B_T)) and they describe the main features of the progressive BD effect on different stacks [190].

**Figure 30:**
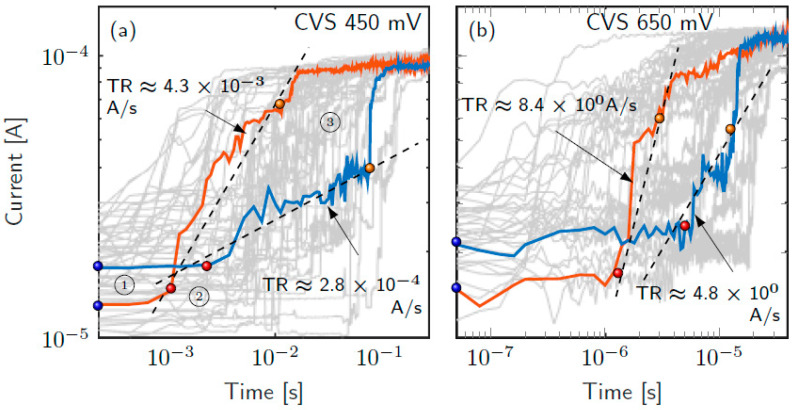
Current transient of the DUTs under constant voltage stress (CVS). (**a**) represents the lowest –450 mV– voltage whereas (**b**) the highest –650 mV–. Ball marker 1 points out the initial current (I_Init_), whereas 2 the onset of the progressive increase of current (I_On_) and 3 the final jump to the compliance level (I_End_).

In this section we considered a RRAM device based on a MIM stack with a 10 nm thick atomic layer deposited HfO_2_ film sandwiched between Ti and TiN electrodes [193]. During the HRS to LRS transition the current (*I_Tr_*) increases gradually with time evidencing the progressive nature of the SET event (see Figure 30a,b). It is a noisy and progressive process well in agreement with the literature [176,177,178,194] whose duration shows a strong voltage dependence and dispersion. The time evolution of the HRS to LRS transition is quantified by the slope *dI_Tr_/dt*, as defined in [169,185,195]. *TR* values were experimentally evaluated through measurements such as those shown in Figure 30a,b (approximately 100 measurements for each voltage value).

**Figure 31:**
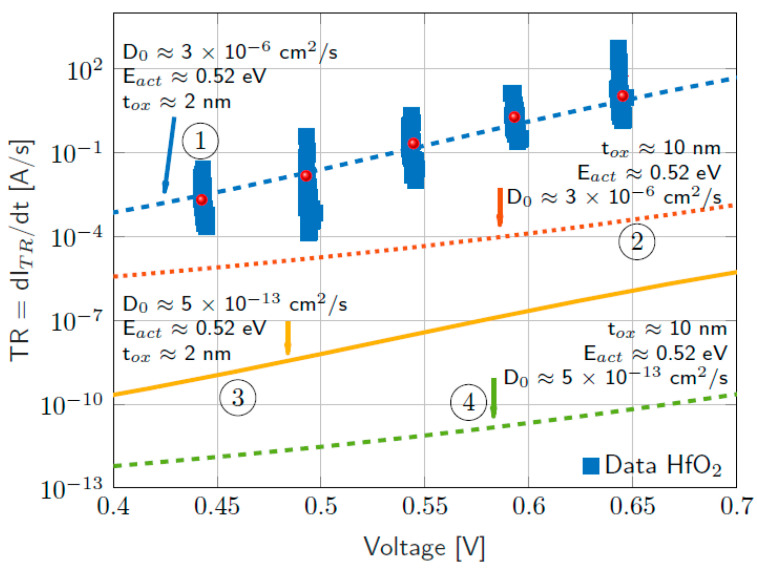
TR data (square markers –ball markers represent the mean value–) fitted assuming oxygen vacancies and t_gap_ = *t_ox_* (cyan dashed line – curve N° 1). Additionally, TR assuming literature values for *t_ox_* and D is plotted –curves N° 2, 3 and 4–. TR presents a strong dependence with applied voltage, increasing almost one order of magnitude for every 50 mV step.

The fitting results for TR as a function of the applied voltage, obtained with the proposed model in Equations (59) and (60), are illustrated in Figure 31. The proposed fit accounts for both the *t_ox_* reduction (*t_ox_* considered is equal to *t_gap_*~2 nm due to the forming step) and the increase in diffusivity (*D_0_* is in the order of ~10^−6^ cm^2^/sec as other species are considered to complete the CF, i.e., oxygen vacancies). The rest of the parameters involved remain as previously mentioned in the literature (*E_act_*~0.3–0.7 eV, *f*_2_~0.1 and *f*_1_~1) [193].

To implement this model for the SET event, the effect of the *t_ox_* reduction after the forming step was considered. First, a forming operation (a controlled dielectric BD) creates the CF through the fresh oxide layer. Then, the switching mechanism is driven by the creation of a gap (RESET) and the restoration of the CF (SET). In the case under study, it has been demonstrated using the statistics of the SET switching time (*t_Set_*) (i.e., the time to complete the HRS to LRS transition) that t_gap_ ≈2 nm is a reasonable value [193].

**Figure 32:**
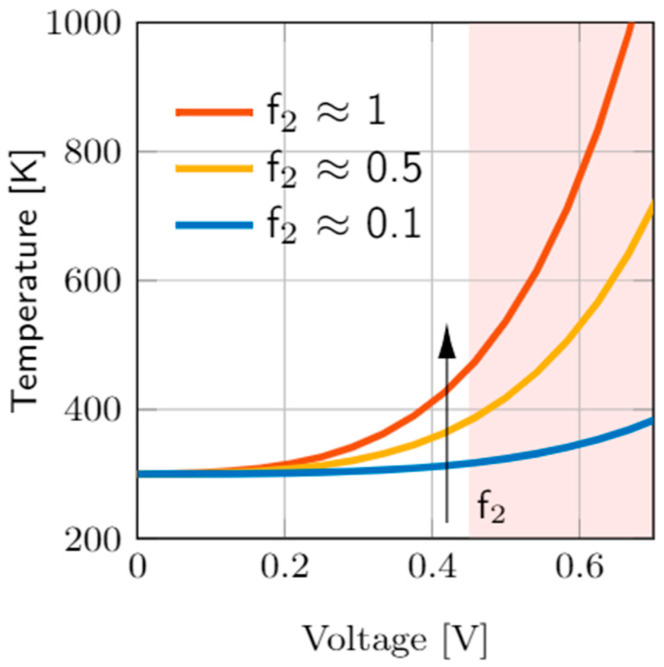
Temperature estimation according to Equation (60) as function of voltage and the energy loss in the CF. The red shaded zone indicates the voltages employed for the CVS (0.45 to 0.65 V).

Figure 32 presents the temperature calculations as a function of voltage provided Equation (60). *f_2_* represents the fraction of energy lost by the carriers, which ranges from 0 to 1. This parameter also depends on the temperature, mainly because of phonon-electron scattering [183]. Therefore, *f_2_* is a function of voltage and temperature whose behavior is found by a best fitting procedure. The influence of *f_2_* on the temperature is shown in Figure 32 for different *f_2_* values.

**Figure 33:**
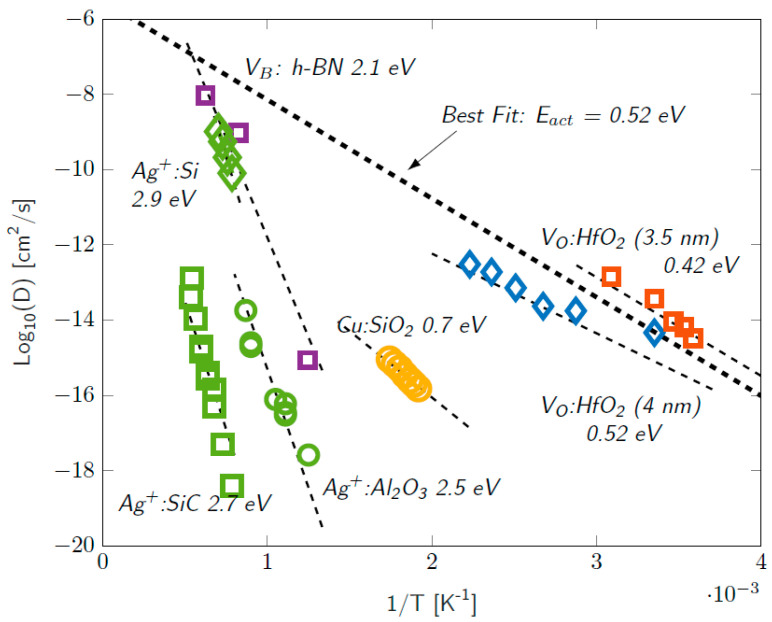
Reported values of diffusivity for different atomic species vs. reciprocal of temperature. The diffusivity D required for TR fitting is shown to be in the same range as the OVs diffusivity. OVs diffusivity [197] is ~10^4^ times higher than for the metallic species as Cu:SiO_2_ [198], Ag:SiC, Ag:Si and Ag:Al_2_O_3_ [199,200]. VB diffusivity in h-BN data corresponds to [201].

The best fitting diffusivity required to reproduce the TR vs. voltage is observed in Figure 33. The data have been calculated assuming that *D_0_* is in the order of 3 × 10^−6^ cm^2^/s and *E_act_* = 0.52 eV as indicated in [193]. In HfO_2_-based RRAM devices, the SET event is explained as the completion of the gap due to the migration of O_2_-ions through a field-assisted and thermally activated effect, which creates the oxygen vacancies that fill the gap along the CF [38,44,53,195,196]. This is quite a relevant point to notice, as the diffusivity of oxygen vacancies (OVs), in a HfO_2_ layer of thickness like *t_gap_* spread over a range similar to the fitted diffusivity for the TR [197] (see Figure 33).

The diffusivity required to fit the experimental data of catastrophic BD in both SiO_2_ and high-k (Al_2_O_3_ or HfO_2_) stacks with metal electrodes are of the order of 10*^−^*^13^ cm^2^/s at 1000 K, with activation energies ranging from 0.3 to 0.7 eV [169], where such values are in a range compatible with the diffusivity of metals in dielectrics (see in Figure 33. the case of Cu diffusion into SiO_2_ layers [198]).

It should be mentioned that the state transition for a non-volatile regime in metal/h-BN/metal stacks (i.e., non-reversible event) has been studied with a similar approach where the best fitting value *E_act_* is also much larger (*E_act_* = 1.3 eV) [184,189]. Such discrepancies may lay on the fact that the particular species involved in the electromigration and/or diffusion process may change, depending on the severity of the SET event (volatile and non-volatile), as it occurs between the BD and SET events in HfO_2_ stacks. While in the BD event in HfO_2_ the diffusing ion species are considered to be the metallic ions from the electrodes (*D_0_* = 1 × 10^−13^ cm^2^/sec, *E_act_* = 0.3–0.7 eV [159,169,189]), the migration of oxygen vacancies from the TMO layer are responsible of the SET transition event (*D_0_* = 1 × 10^−6^ cm^2^/s, *E_act_* = 0.52 eV) [188].

To make clear the impact of both the *t_ox_* reduction and the diffusivity increase (*D_0_*), alternative fitting values were used to plot curves N° 2, N° 3 and N° 4 in Figure 31. Curve N°4 coincides with the TR for gate-oxide BD, as it considers diffusivity of metals in oxide layers and no *t_ox_* reduction. The comparison of curves N° 1 and N° 4 evidence that TR is significantly higher than the TR expected for the voltage range considered. It is important to point out that TR calculated considering only a *t_ox_* reduction or an increase in diffusivity (*D_0_*) cannot meet the experimental data. This can be interpreted as that the two factors determine the dependence with the TR voltage, since none of them can separately adjust the results independently.

## 6. Conclusions

A comprehensive and exhaustive revision of RRAM thermal models is presented. Different approaches have been considered and described, including different conductive filament geometries, operation regimes, filament lateral heat losses, several temperatures to characterize each conductive filament, etc. A 3D numerical solution of the heat equation within a RRAM simulator was used. In addition, analytical models have been developed using equations describing the relevant physics behind the heat equation accounting for steady-state and non-steady-state device operation. A general memristor modeling framework was formulated considering the temperature as a state variable. Moreover, the quantum perspective was included in a mesoscopic context; this is a must due to the nanometric dimensions of the devices under study. In this respect, thermal effects were considered in the formulation of the quantum point contact model. Finally, conductive filament thermometry in usual RRAM technology was studied in detail. Since the physics underlying resistive switching is mainly based on thermally activated physical mechanisms, an accurate description of thermal effects is essential. As far as we know, there are not similar manuscripts that put together these types of effects under one roof.

## Figures and Tables

**Table 1:** Verilog-A implementation of some of the thermal models described.

Thermal Model	Verilog-A Code for the Temperatures Calculation
**TM1**	*T* = *T_0_* + sigma*V**2/(8.0*kth);
**TM2**	E = V/tox; alpha = tox/2.0*sqrt(2*h/(kth*rcf)); *T* = *T_0_* + (sigma*E**2*rcf*(exp(alpha)−1)**2)/(2*h*(exp(2*alpha)+1));
**TM3**	LCF = tox−g; rg = sqrt(rt*rb); eta = rt/rb; E = V/LCF; alpha = LCF*sqrt(2*h/(kth*rg)); *T* = *T_0_* + rg*sigma*E**2/(eta*h)*(0.5 − (exp(alpha/2.0)/(exp(alpha) + 1));
**TM4**	analog function real fdt_0_; real sigmat,rcf,E,alpha; input sigmat,rcf,E,alpha; begin fdt_0_ = sigmat*rcf*E**2*tanh(alpha*tox/2.0)/(sqrt(2*kth*h*rcf)); end endfunction analog function real fT; real sigmat,rcf,eta,alpha,dt_0_; input sigmat,rcf,eta,alpha,dt_0_; begin fT = *T_0_* + sigmat*rcf*E**2/(2*h)*(1-cosh(alpha*tox/2.0)) + dt_0_/alpha*sinh(alpha*tox/2.0); end endfunction analog function real falpha; real rcf; input rcf; begin falpha = sqrt(2*h/(kth*rcf)); end endfunction analog function real fsigmat; real T; input T; begin fsigmat = sigma/(1 + alphat * (*T* − *T_0_*)); end endfunction

**Table 2:** Model parameters employed for the simulations performed with the Stanford model, in particular for Figure 12 and Figure 13.

Stanford-PKU Model Parameters			
Device Parameters	Unit	Resistive Switching	
		SET	RESET
*V_o_*	V	0.4	
*I_0_*	mA	0.2	
*g_0_*	nm	0.35	
*ν_0_*	m/s	10^6^	
*α*	-	1	
*β*	-	3	
*γ_0_*	-	10	

## Data Availability

The datasets generated during and/or analysed during the current study are available from the corresponding author on reasonable request.
